# Wild barley cytoplasms reduce grain weight plasticity, with environment-dependent cytonuclear epistasis at the *ari-e* locus

**DOI:** 10.1007/s11032-026-01673-6

**Published:** 2026-05-19

**Authors:** Schewach Bodenheimer, Avital Beery, Shaharit Ziv, Abelina Fuentes, Solar Parry, Jonathan Berlingeri, Lamis Abdelhakim, Klára Panzarová, Christine Diepenbrock, Eyal Fridman

**Affiliations:** 1 The Institute of Plant Sciences, Agricultural Research Organization, Volcani Institute, Rishon LeZion, 7505101 Israel; 2 The Robert H. Smith Faculty of Agriculture, Food and Environment, The Hebrew University of Jerusalem, Rehovot, 76100 Israel; 3 Department of Plant Sciences, University of California, Davis, USA; 4 PSI (Photon Systems Instruments), spol. s.r.o., Drásov, Czech Republic

**Keywords:** Crop wild relatives, Cytonuclear interaction, Barley, *ari-e*, Phenotypic plasticity

## Abstract

Although cytoplasmic genetic variation influences agronomic traits, its potential for breeding applications remains unexplored. We validated cytoplasmic effects on grain traits in barley and tested their portability across nuclear backgrounds and environments. The cytonuclear multi-parent population validation panel (CMPPV) and Magal reciprocal validation panel (MRVP) were evaluated in replicated field trials across two thermally contrasting Israeli environments. The wild B1K-50-04 cytoplasm consistently increased thousand grain weight (TGW) by 6–11% and grain width (GW) by 2–3% relative to the corresponding cultivated cytoplasm within each reciprocal comparison, explaining up to 9.5% of TGW, and 8% of GW variance. These effects persisted in segregating populations derived from crosses with the elite cultivar Magal, demonstrating portability across nuclear backgrounds. Three-way analysis of marker × cytoplasm × environment interactions across a targeted set of loci identified a chromosome 5 H cluster of significant marker effects, including genotype × cytoplasm × environment signals near the *ari-e* locus; the hypothesized segregation of an *HvDep1* mutant allele was confirmed by Sanger sequencing. Near-infrared spectroscopy analysis of grain composition revealed environment-dependent cytonuclear epistasis for the *ari-e.GP* allele, increasing protein content only in the B1K-50-04 cytoplasmic background at one field site. Image-based high-throughput phenomics under controlled conditions identified morphological and canopy spectral reflectance indices that diverged between cytoplasms and were correlated with final grain dimensions, suggesting that taking cytoplasmic variation into account can reveal novel trait relationships. Findings demonstrate that wild cytoplasms offer portable genetic resources for crop improvement, and that the utility of major nuclear loci in breeding can depend on cytoplasmic context.

**Supplementary Information:**

The online version contains supplementary material available at 10.1007/s11032-026-01673-6.

## Introduction

Cytoplasmic genetic variation, encoded by mitochondrial and chloroplast genomes, influences a broad range of agronomic traits in plants beyond the well-characterized cytoplasmic male sterility systems (Chase 2007; Melonek et al. 2021). In *Arabidopsis thaliana*, reciprocal cross analyses showed that more than half of the examined metabolites display significant maternal-based heritability (Joseph et al. 2013), and field evaluations of *Arabidopsis* cytolines demonstrated that most adaptive traits exhibit significant interactions between organellar and nuclear genomes (Roux et al. 2016). Evidence from crop species supports these findings. In maize, alloplasmic lines created by introducing *teosinte* cytoplasms into a maize nuclear background differed markedly from the recurrent parent for a wide range of developmental and physiological traits, highlighting the contribution of cytoplasmic backgrounds to phenotypic variation (Allen 2005). Similarly, in *indica* rice, nuclear substitution lines that shared an identical nuclear genome but carried contrasting cytoplasms exhibited significant cytoplasmic effects on thousand grain weight (TGW) across environments, and a significant three-way interaction between nucleus, cytoplasm, and environment for filled-grain ratio (Tao et al. 2011). While the adaptive nature of cytoplasmic polymorphisms is debated (Bock et al. 2014), several lines of evidence support non-neutrality. These include the discovery of large-effect mutations such as Ser-264-Gly in the chloroplast *psbA* gene, which confers triazine herbicide resistance to a wide variety of weed species (Powles and Yu 2010). Cytoplasm-driven local adaptation has also been described in *Helianthus* species (Sambatti et al. 2008). In Geraniaceae, substitution rates in plastid-encoded RNA polymerase subunits track those of their nuclear sigma factor partners, pointing to cytonuclear coevolution (Zhang et al. 2015). Cytonuclear interactions have also been shown to modulate the plasticity of photosynthetic rhythmicity and growth in wild barley (Tiwari et al. 2024). Together, these findings support adaptive value for cytoplasmic and cytonuclear variation.

While these studies have established the contribution of cytonuclear interactions to phenotypic variation, identifying the specific nuclear loci involved requires dedicated mapping approaches. Using reciprocal F_2_ populations in maize, a joint cytonuclear interaction QTL mapping approach identified six epistatic cytonuclear QTL for plant height and five for ear height, with QTL × cytoplasm interactions explaining up to 18% of phenotypic variance (Tang et al. 2013). In *Saccharomyces cerevisiae*, genetic loci exhibiting mitonuclear epistasis were detected by mapping mitochondrial DNA stability using a multi-parent advanced intercross population, wherein each nuclear genotype was paired with multiple mitotypes (Nguyen et al. 2023). In *Arabidopsis*, cytoplasm significantly affected proline accumulation, accounting for 10.6% of total variation, and QTL x cytoplasm interactions were found at two separate loci, affecting growth rate and water-use efficiency under dry conditions, respectively (Lovell et al. 2015). These studies demonstrate that experimental designs involving reciprocal crosses or structured populations with controlled cytoplasmic inheritance can effectively localize nuclear loci involved in cytonuclear epistasis.

Barley (*Hordeum vulgare* L.) provides a proven system to investigate cytonuclear complexities in an agricultural set-up (Bodenheimer et al. 2025). New evidence suggests that the earliest domestication trends for barley go back 25,000 years (Guo et al. 2025), with its wild ancestor (*H. vulgare* ssp. *spontaneum*) harboring substantial ecogeographic adaptation and genetic diversity (Hübner et al. 2009; Russell et al. 2016). Systematic studies of maternal inheritance in barley date back more than 90 years, when chlorophyll-deficient phenotypes were shown to be exclusively transmitted through the female parent (Robertson 1937). Subsequent studies using chloroplast substitution lines showed that cytoplasm diversity contributes to variation in productivity traits depending on the nuclear donor cultivar (Goloenko et al. 2002), while reciprocal crosses between wild and cultivated germplasm revealed cytonuclear interactions affecting circadian clock plasticity and growth dynamics (Bdolach et al. 2019; Tiwari et al. 2024). Building on these foundations, the cytonuclear multi-parent population (CMPP), comprising 951 doubled haploid lines from crosses between 10 wild accessions and the elite cultivar Noga, was developed to systematically dissect barley cytonuclear epistasis. Using a specialized mapping approach, 16 cytonuclear quantitative trait loci (cnQTL) controlling various grain and spike traits were identified, and Shukla’s stability variance revealed that the B1K-50-04 wild cytoplasm substantially improved TGW stability across environments (Bodenheimer et al. 2025). Notably, the B1K-50-04 allele of the plastidial *RpoC1* gene was also shown to modulate thermal plasticity in transformed *Arabidopsis* lines (Tiwari et al. 2024), suggesting that specific cytoplasmic factors can buffer phenotypic expression across environments. Environment-dependent effects are similarly well-documented for nuclear loci controlling grain traits in barley. A prominent example is the *ari-e* semi-dwarfing locus on chromosome 5 H, where the causal gene *HvDep1* pleiotropically affects plant height, TGW, grain length (GL), and heading date (Wendt et al. 2016; Dang et al. 2020). Multienvironment field trials demonstrated that allelic effects at this locus on grain yield range from negative to positive depending on environmental conditions (Wendt et al. 2016). The *ari-e.GP* allele, originally induced in cv. Golden Promise (Dockter and Hansson 2015), has since been widely deployed in barley breeding programs (Fox and Lance 2020).

Here, we followed up on the CMPP study (Bodenheimer et al. 2025) in three ways. First, we generated the Magal reciprocal validation panel (MRVP), which consists of four reciprocal F₂ populations that place CMPP-derived cytoplasms into a segregating cv. Magal nuclear background. This design tests whether wild cytoplasmic effects are portable to an independent elite background and allows for direct genotype × cytoplasm × environment (G × C × E) testing. Second, we used a targeted marker panel to revisit previously identified cnQTL in the new background. Third, we used near-infrared spectroscopy (NIRS) to test whether cytonuclear effects extend beyond grain size and weight to compositional traits, and the PlantScreen phenomics platform to determine whether the cytoplasmic effects observed for grain traits in two contrasting CMPP subfamilies extend to vegetative growth and canopy physiology. Alongside the MRVP, we evaluated the CMPP validation panel (CMPPV), a subset of four CMPP subfamilies retaining the original ‘Noga’-based nuclear background, across two contrasting Israeli field environments.

## Materials and methods

### Plant material

**Fig. 1:**
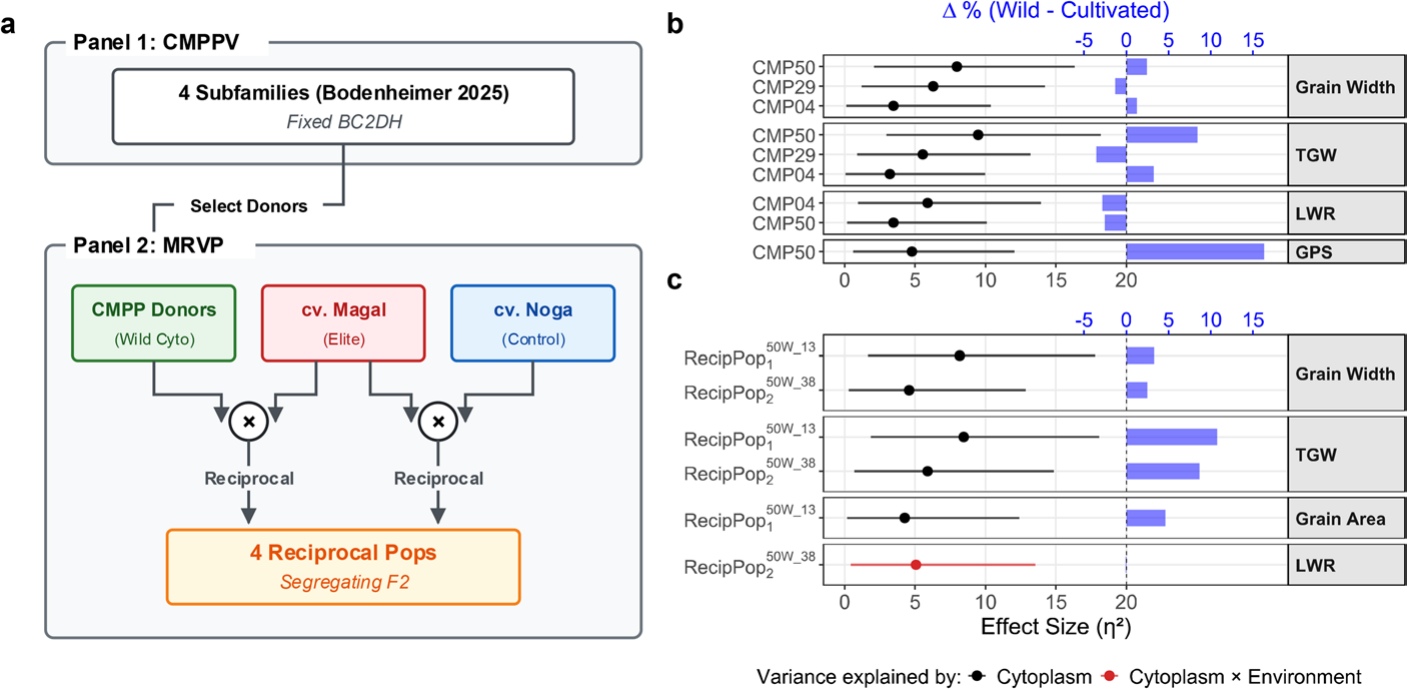
Panels design, and variance explained by cytoplasm and cytoplasm × environment. (**a**) Panel 1 shows the CMPP validation panel (CMPPV), comprising four fixed BC_2_DH subfamilies used to select donor lines carrying wild cytoplasms. Panel 2 shows the Magal reciprocal validation panel (MRVP), generated by reciprocal crosses between selected CMPP donors and cv. Magal, with cv. Noga included, producing four segregating F_2_ reciprocal populations for validation of cytoplasmic effects and analysis of cytonuclear interactions. (**b**) Significant terms within CMPPV populations, and (**c**) MRVP populations, derived from two-way ANOVA. Blue bars show percentage increase (Δ%) of the wild cytoplasm compared to its cultivated counterpart for the respective traits. TGW, thousand grain weight; LWR, length-to-width ratio; GPS, grains per spike

The CMPPV included four subfamilies (CMP04, CMP09, CMP29, and CMP50) that were selected from the original CMPP based on significant cytoplasmic effects on grain traits (Bodenheimer et al. 2025). Each subfamily consisted of BC_2_DH lines derived from reciprocal crosses between wild barley (*Hordeum vulgare* ssp. *spontaneum*) donors and the Israeli cv. Noga. The MRVP consisted of four reciprocal F₂ populations: RecipPop_1_^50W_13^, RecipPop_2_^50W_38^, RecipPop_3_^04W_06^, and RecipPop_4_^Noga^. These populations were produced by crossing the Israeli two-row cv. Magal, which carries the cytoplasm of the Australian cv. Hindmarsh (E. Fridman and Agridera Seeds & Agriculture Ltd., personal communication), reciprocally with three CMPP donor lines (CMP50W_13, CMP50W_38, CMP04W_06) and with ‘Noga’, respectively. This design introduced three distinct cytoplasms into F₂ nuclear backgrounds segregating for ‘Magal’ and donor alleles: the B1K-50-04 and B1K-04-04 cytoplasms (Hübner et al. 2009; Bodenheimer et al. 2025), and the cultivated ‘Noga’ cytoplasm (Fig. 1a, Online Resource 1).

### Cytoplasm nomenclature

Throughout this manuscript, wild cytoplasms are referred to by their original donor accession (B1K-50-04, B1K-04-04, B1K-29-13, or B1K-09-03; Fig. 1a, Online Resource 1); cultivated cytoplasms are referred to by the maternal parent contributing the cytoplasm (‘Noga’ in the CMPPV; ‘Magal’ in the MRVP).

### Field trials

Field trials were carried out during the 2023–2024 growing season at two sites: Yotveta (29°53’13.6” N, 35°04’35.4” E) and Mibhor (31°37’51.2” N, 34°45’31.0” E). The CMPPV was evaluated in 0.3-m^2^ mini-plots of 60 plants, using a completely randomized design with 3 randomly replicated mini-plots per line in each environment, and 10 representative spikes were harvested per mini-plot for grain trait measurements. For the MRVP, which was planted in the same field alongside the CMPPV, a randomized complete block design was used with 8 blocks, each one containing 18 mini-plots. Owing to the segregating structure of this reciprocal panel, each mini-plot consisted of seven individually identifiable plants, which were sampled for genotyping at the three-leaf stage.

### Grain phenotypic measurements

Small-scale threshing of spikes was performed using an RC52 rice-milling machine (Yamamoto Electric, Fukushima, Japan). For the MRVP, grain morphology and count were measured using a Marvin ProLine II seed analyzer (MARViTECH GmbH, Wittenburg, Germany) to determine grain area (GA), GL, grain width (GW), length-to-width ratio (LWR), and TGW. For the CMPPV, GA, GL, and GW were quantified using a Marvin ProLine seed analyzer (MARViTECH) with percentiles (representing frequency distributions) for each sample reported in fractional bins for each trait. Average GA, GL, and GW for each sample were then determined via a weighted average of the upper and lower bounds of the bin in which the 50th percentile for each trait was reached (assuming linear increases across that bin; this approach was validated with ~ 130 samples; Online Resource 2). Number of grains per spike (GPS) were determined in the CMPPV by counting and averaging grains from harvested spikes (Online Resource 3). For MRVP populations, grains per plant (GPP), above-ground biomass, and total grain weight per plant were additionally recorded (Online Resource 4).

### Shoot phenotypic measurements

To investigate the contrasting cytoplasmic effects on morphological and physiological traits at the canopy level, a high-throughput phenotyping trial was conducted on the CMP29 and CMP50 populations. Two seedlings were transplanted in 5-L pots filled with Klassman 2 peat mixed with sand in 3:1 proportion, and blue holders were inserted to support plants and avoid biomass overlapping. Plants grew in the greenhouse under a 14 h/10 h day/night photoperiod at 310 µmol m^−2^s^− 1^ with relative humidity of 45/30% day/night, and were acclimated for 1 week prior to phenotyping. Plants were phenotyped using the PlantScreen modular phenotyping platform (Photon Systems Instruments, Drásov, Czech Republic) from the tillering stage (7 days after transplanting) until the end of the stem-elongation stage (49 days after transplanting). Multiple imaging sensors were used, including chlorophyll fluorescence, RGB morphology, thermal infrared, and hyperspectral imaging modalities (Online Resource 5), resulting in the capture of 126 phenotypic traits followed by automatic image analysis as described in Abdelhakim et al. (2024). Chlorophyll fluorescence imaging protocols were performed as described previously (Tietze et al. 2025).

### NIRS acquisition

Grain macronutrient contents of protein, ash (total minerals), moisture, and fat were quantified as percentage of grain composition using a benchtop NIRS instrument (DS2500; FOSS, Eden Prairie, MN, USA). Spectral data were acquired over the wavelength range of 400 to 2500 nm at 0.5-nm intervals using the pre-existing Cereals/Grains calibration available from the instrument manufacturer (O.N. 60070685), and recorded in log (1 R^− 1^) format (ISIscan Nova, FOSS). For the 431 MRVP samples with sufficient sample mass, 25 g of whole grain was scanned using a FOSS large sample cup, and these samples were used to develop the custom calibration described in the next section. An additional 239 MRVP samples that had < 25 g of whole grain available were also scanned, using the entire sample mass that was available for each respective sample.

Predicted values for protein, fat, ash, and moisture along with secondary parameters (such as global and neighborhood H) from the pre-existing Cereals/Grains calibration were exported to a Microsoft Excel (.xlsx) file in FossManager. Distributions of H values are presented in Online Resource 6. Raw spectral data were exported as NIRS (.nir) files in FossManager and converted to comma-separated values (.csv) format using Spectragryph software (F. Menges, Version 1.2.16.1), after which all spectral preprocessing and statistical analyses were performed.

A subset of 60 samples (30 from each location) from the initial 431 samples was selected using the Kennard–Stone algorithm to be sent for reference wet-chemistry analyses. Sample selection was performed using the “kenStone()” function in the R package “prospectr” (Stevens and Ramirez-Lopez 2025) with metric set to ‘mahal’, i.e., the Mahalanobis distance in the principal component (PC) space based on the spectral data. PCs explaining 99% of the cumulative spectral variance were retained for sample selection (PC = 0.99). These samples were ground in a tube mill (IKA Works, Wilmington, NC, USA) using multiuse milling tubes (IKA MMT 40.1; IKA Works), stored at −20 °C until day of scanning, and equilibrated to room temperature for approximately 30 min prior to NIRS scanning of 25 g whole-grain flour. Ground samples were then stored at −20 °C and submitted for reference wet-chemistry analyses at the UC Davis Analytical Laboratory for protein (AOAC Official Method 990.03), fat (AOAC Official Method 2003.05), ash (AOAC Official Method 942.05), and dry matter (oven drying at 105 °C for 3 h), which was used to calculate moisture.

### NIRS custom-calibration development

Custom calibration for the NIRS was developed using the wet-chemistry data as reference values. Spectral preprocessing included testing the use of standard normal variate (SNV) correction, multiplicative scatter correction, detrending, baseline correction, and Savitzky–Golay (SG) smoothing and its first and second derivatives (SG1 and SG2, respectively). Combined pretreatments (such as SNV + detrending, or SNV + SG1) were also evaluated. All spectral preprocessing was conducted using the “prospectr” package in R (Stevens and Ramirez-Lopez 2025). Spectral outlier detection was performed using PC analysis (PCA). For each spectral pretreatment, PCA was conducted using the “prcomp()” function in base R with scale set to TRUE, and scores from the first five PCs were retained. Mahalanobis distances were calculated from the PCA scores using a minimum covariance determinant estimator. Samples exceeding the 99.5th percentile were flagged as spectral outliers for a given pretreatment. Samples identified as outliers in at least 75% of the pretreatments were classified as global spectral outliers and removed from subsequent analyses, but no samples met this threshold for global removal. Outliers in wet-chemistry reference data were identified on a trait-by-trait basis using the interquartile range (IQR), where values falling below Q1–1.5 x IQR or above Q3 + 1.5 x IQR were classified as outliers and replaced with missing values prior to calibration development, while retaining other non-outlier trait values for the same sample. Partial least squares regression models were developed using the “pls” package in R (Liland et al. 2024). For each trait and pretreatment combination, five iterations of 5-fold cross-validation were conducted with random assignment of samples to folds. For each iteration, the optimal number of PCs was initially determined using the one-sigma heuristic based on root mean square error of prediction (RMSEP). RMSEP scree plots were visually inspected for each model to confirm or adjust the number of components when necessary (e.g., for an overly parsimonious model). Predictions were then generated using the “predict()” function with that optimized number of components. Model performance across spectral pretreatments was then compared using RMSEP and the Pearson correlation between predicted and observed values, averaged across five iterations. The combined spectral pretreatment of SNV + SG demonstrated reasonably high accuracy and reasonably low standard deviation for all traits and iterations, and was selected for development of the custom calibration. For each trait, the number of PCs selected in the fifth iteration was the number used in the custom calibration.

Predicted values from the custom calibration and from the pre-existing Cereals/Grains calibration were compared against wet-chemistry reference values, and the coefficient of determination (R^2^) was calculated and reported (Online Resource 7). R² values increased markedly for all four traits, ash (0.44 to 0.81), fat (0.16 to 0.94), protein (0.63 to 0.93), and moisture (0.19 to 0.81), indicating substantially improved model fit (Online Resource 7). As a supplementary examination of model performance, an 80/20 train–test split was generated by selecting the test set using the Kennard–Stone algorithm on the Mahalanobis distance calculated from the first two PCs of the pretreated spectra. Model performance under this sampling scheme was consistent with cross-validation results and did not affect pretreatment selection. The finalized calibration, developed based on the initial 431 MRVP samples (and namely, the subset of 60 samples selected from the 431 and sent for wet-chemistry reference analyses), was subsequently applied to the SNV + SG-pretreated spectra of the 239 MRVP samples that had < 25 g of available grain to generate predicted trait values without further model recalibration. Similar outlier detection was performed, and two samples met the ≥ 75% frequency threshold for global removal. All spectral preprocessing, PCAs, outlier detection, and chemometric analyses were conducted in R (Version 4.3.2).

### Derived compositional mass traits

To capture variation in the absolute amounts of grain macronutrients, as opposed to their relative concentrations, we computed six derived mass traits by combining NIRS-predicted composition with weight-based measurements. A dry matter fraction was first calculated for each sample as.

1 $$\:DM\:=\:\frac{100\:-\:Moisture}{100}$$

Three per-grain traits (Prot1000, Fat1000, Ash1000; in g per 1000 grains) were then obtained as.

2 $$\:{Trait}_{1000}\:=\:\frac{TGW\:\times\:\:DM\:\times\:\:C}{100}$$

where *C* is the NIRS-predicted concentration of protein, fat, or ash (% dry basis). Analogously, three per-plant yield traits (ProtP, FatP, AshP; in g per plant) were derived as.

3 $$\:{Trait}_{P}\:=\:\frac{GWP\:\times\:\:DM\:\times\:\:C}{100}$$

These derived traits integrate grain size or yield with nutritional composition and were included alongside the original morphological and compositional traits in all downstream analyses of the MRVP.

#### Cytonuclear interaction analysis

For the CMPPV (fixed lines), phenotypic values were aggregated into a single mean for each line within each environment. For the MRVP, where each individual represents a unique genotype distinct from its neighbors, single-plant measurements were utilized directly in the linear models. Univariate outliers were identified and removed within each environment × population combination using the boxplot method with method = “asymmetric” and *k* = 1.5, implemented via the boxB function from the univOutl package v.0.4 (D’Orazio 2022).

Cytoplasmic effects were assessed using two-way ANOVA for each population, fitting the model:

4 $$\:{y}_{ijk}=\mu\:+{C}_{i}+{E}_{j}+(C\times\:E{)}_{ij}+{\epsilon\:}_{ijk}$$

where $\:{y}_{ijk}$ is the trait value, $\:\mu\:$ is the overall mean, $\:{C}_{i}$ is the cytoplasm effect, $\:{E}_{j}$ is the environment effect, $\:(C\times\:E{)}_{ij}$ is the cytoplasm-by-environment interaction, and $\:{\epsilon\:}_{ijk}$ is the residual error. Type III ANOVA was performed using the car package v.3.13 (Fox et al. 2024). Eta squared (η²) values, representing the proportion of total phenotypic variance attributable to each effect, and associated confidence intervals (CIs) were calculated using the effectsize package v.1.0.1 (Ben-Shachar et al. 2025). A term was treated as significant if it satisfied *p* ≤ 0.05, η² ≥ 1%, and lower CI bound > 0 (Online Resource 8). Cytoplasmic effect magnitudes were expressed as percentage change (Δ%), calculated as the mean across environments of $\:100\times\:({\mu\:}_{Wild}-{\mu\:}_{Cultivated})/{\mu\:}_{Cultivated}$, where $\:\mu\:$ represents the trait mean within each environment.

### Quantification of cytoplasmic divergence

To analyze the phenomics data from the CMP29 and CMP50 populations, we first reduced the dimensionality of the PlantScreen phenotypic dataset using correlation-based pruning (*r* = 0.95), reducing the number of traits from 126 to 66 (Online Resource 9). For each retained trait, Cohen’s d effect sizes were calculated by comparing wild vs. ‘Noga’ cytoplasms within each population and measurement day using the effectsize package (Ben-Shachar et al. 2025), then weighting by the proportion of significant measurement days (*p* < 0.05, two-sample t-tests). Divergence between CMP29 and CMP50 was calculated as the absolute difference in weighted effect sizes. Traits exceeding the 90th percentile of divergence (threshold = 0.16; Online Resource 10) were classified as significantly divergent (Fig. 3a, Online Resource 9).

### Genotyping and G × C × E analysis

Genotyping of the MRVP was conducted by LGC (Hertfordshire, UK) using a targeted set of 35 Kompetitive Allele Specific PCR (KASP) markers (Online Resource 11) selected from marker–trait associations (MTAs) and cnQTL previously identified in the CMPP study (Bodenheimer et al. 2025). After removing markers deviating significantly from the expected 1:2:1 Mendelian segregation ratios (χ² test, *p* < 0.01), a three-way ANOVA was performed for each population to test for marker × cytoplasm × environment (G × C × E) interactions:

5 $$\:{y}_{ijkl}=\mu\:+{G}_{i}+{C}_{j}+{E}_{k}+{\left(GC\right)}_{ij}+{\left(GE\right)}_{ik}+{\left(CE\right)}_{jk}+{\left(GCE\right)}_{ijk}+{\epsilon\:}_{ijkl}$$

where $\:G$ is the marker genotype, $\:C$ represents cytoplasm class (‘Magal’ vs. the alternative cytoplasm: wild for RecipPop_1–3_, ‘Noga’ for RecipPop_4_, see Fig. 1a, Online Resource 1), and $\:E$ is environment (Mibhor vs. Yotveta). Effect sizes for the three-way analysis were extracted using the effectsize package (Ben-Shachar et al. 2025) and quantified as η² (Online Resource 12), with 95% CIs computed using the same package. Terms were reported as significant when *p* ≤ 0.05, η² ≥ 1%, and the lower 95% CI bound for η² > 0.

### Candidate gene validation

The *ari-e.GP* allele (Wendt et al. 2016) was validated by amplifying the second exon of *HvDep1* (HORVU.MOREX.r3.5HG0480200) from ‘Magal’ and CMP50W_38. Amplification was carried out with forward primer 13A7 (5’-GGCAACAACTTTGCATGGCCGA-3’) and reverse primer 12I9 (5’-GAGCTAGTCCCGTCAAGTCC-3’). PCRs were run with DreamTaq Green PCR Master Mix (Thermo Fisher Scientific, Waltham, MA, USA) using a touchdown protocol that started at 65 °C and decreased by 0.3 °C per cycle for 35 cycles. Amplicons were then precipitated and sequenced by standard Sanger sequencing.

## Results

### The B1K-50-04 cytoplasm consistently increases TGW and GW

Having previously identified cytoplasmic effects on grain traits in the CMPP, most notably the TGW-increasing effect of the B1K-50-04 cytoplasm (Bodenheimer et al. 2025), we asked whether these effects are robust across environments and transferable to other elite genetic backgrounds. We developed two validation panels: the CMPPV and the MRVP (Fig. 1a, Online Resource 1; see Methods), which were evaluated in two contrasting environments (Mibhor and Yotveta; Online Resource 13). While the CMPPV represents 40% of the original CMPP (4 out of 10 donors), the MRVP introduces reciprocal crosses between wild-cytoplasm-carrying CMPP lines and the smaller-grained cv. Magal (Online Resource 14, Online Resource 15).

For each population in the two panels, we fitted a two-way ANOVA with cytoplasm and environment as factors, and quantified effect sizes as η² (see Methods). In the CMPPV, cytoplasm explained 9.47% of the phenotypic variance for TGW in CMP50, 5.54% in CMP29, and 3.19% in CMP04 (Fig. 1b), values that in part aligned with, or exceeded the 4.8% cytoplasmic contribution to TGW previously reported for the full CMPP (Bodenheimer et al. 2025). Importantly, the directionality of the cytoplasmic effect was also consistent with our previous study, with the B1K-50-04 and B1K-04-04 cytoplasms both causing a relative increase in TGW (+ 8.44% and + 3.23%, respectively) compared to the ‘Noga’ cytoplasm, whereas the B1K-29-13 cytoplasm yielded a relative decrease in TGW (−3.57%; Fig. 1b). TGW in the CMPPV was strongly driven by GW (R² = 0.71; Online Resource 16). Accordingly, the cytoplasmic effects on GW mirrored those on TGW and replicated our earlier findings: the B1K-29-13 cytoplasm decreased GW by 1.34% (η^2^ = 6.29%), while the B1K-50-04 cytoplasm increased it by 2.44% (η^2^ = 7.96%). Interestingly, while increasing TGW, the B1K-50-04 cytoplasm also positively influenced GPS (η² = 4.77%, + 16.31%). Trial quality data (Online Resource 17) showed that cross-site reproducibility of GPS was comparable to that of TGW (R² = 0.258 vs. 0.290 for Mibhor and Yotveta, respectively), but environment-wise CVs were notably higher (19.31–25.97% vs. 9.73–10.95%, respectively). Thus, despite the GPS effect being supported by consistent line differences across environments, its higher dispersion calls for more cautious interpretation compared to grain-size traits, particularly GW (R² = 0.395, CV = 3.45–3.96%).

Having established that the B1K-04-04, B1K-29-13, and B1K-50-04 cytoplasmic effects are replicated in the CMPPV, we next evaluated whether they persist when transferred to a different nuclear background. Here, we utilized the MRVP, comprised of four reciprocal F₂ populations (RecipPop_1_^50W_13^, RecipPop₂^50W_38^, RecipPop₃^04W_06^, and RecipPop₄^Noga^) that introduce wild or cultivated cytoplasms into the background of ‘Magal’, a modern Israeli two-row variety (Fig. 1a, Online Resource 1). In addition to the segregating cytoplasms, these populations share a complex nucleotype consisting of 50% ‘Magal’ and 50% donor genome, either ‘Noga’ (RecipPop₄^Noga^) or ‘Noga’ with residual wild introgressions (RecipPop_1_^50W_13^, RecipPop₂^50W_38^, and RecipPop₃^04W_06^). Notably, significant cytoplasmic effects were detected only in RecipPop_1_^50W_13^ and RecipPop₂^50W_38^, both carrying the B1K-50-04 cytoplasm (Fig. 1c). In these populations, cytoplasm explained 8.45% and 5.89% of TGW variance, respectively, with the wild cytoplasm increasing TGW by + 10.74% and + 8.67%. Comparable trends were observed for grain size components: GA was significantly increased in RecipPop₁^50W_13^ (η² = 4.25%, + 4.61%), while GW was elevated in both RecipPop₁^50W_13^ and RecipPop₂^50W_38^ (η² = 8.16% and 4.58%, + 3.30% and + 2.48%, respectively). A single cytoplasm × environment interaction was detected for LWR in RecipPop₂^50W_38^ (η² = 5.07%, −0.08%), indicating environment-dependent cytoplasmic modulation of grain shape (Fig. 1c). In contrast, RecipPop₃^04W_06^ and RecipPop₄^Noga^ showed no significant cytoplasmic main effects, suggesting a limited contribution of the B1K-04-04 wild and ‘Noga’ cytoplasms to trait variation in the given setting.

**Fig. 2:**
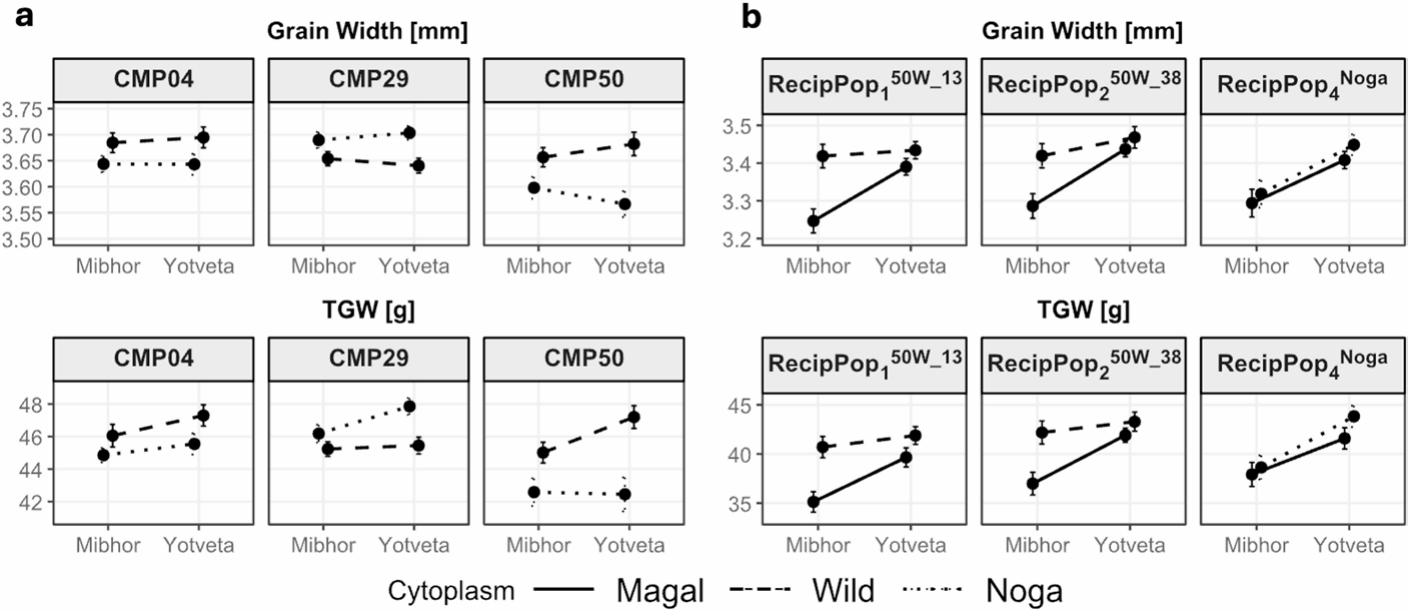
Reaction norms for trait-population combinations with significant cytoplasmic effects. (**a**) CMPPV, (**b**) MRVP

To better understand the environmental dependency of cytoplasmic effects, we examined reaction norms for GW and TGW across the two field sites (Mibhor and Yotveta) in populations showing significant cytoplasmic contributions (Fig. 1). In the CMPPV, cytoplasmic differentiation, i.e., the phenotypic gap between wild and cultivated cytoplasm means within a site, was stronger under Yotveta conditions (Fig. 2a), with the B1K-50-04 cytoplasmic advantage for TGW in CMP50 increasing from + 5.7% at Mibhor to + 11.2% at Yotveta. A similar fold increase was observed for GW between the sites (+ 1.6% and + 3.2%, respectively). This pattern of Yotveta-driven amplification extended to CMP29, where the negative effect of the B1K-29-13 cytoplasm on TGW likewise intensified at Yotveta (from − 2.1% to −5%), whereas the B1K-04-04 cytoplasm showed a site-independent TGW increase (+ 2.7% and + 3.8% for Mibhor and Yotveta, respectively).

The MRVP, on the other hand, revealed an inverted environmental architecture, with the same B1K-50-04 cytoplasm showing its strongest effects at Mibhor rather than Yotveta (Fig. 2b). In both RecipPop₁^50W_13^ and RecipPop₂^50W_38^, the B1K-50-04 cytoplasm conferred substantial TGW advantages at Mibhor (+ 15.9% and + 14.1%, respectively) that diminished 3- to 4-fold at Yotveta. The same site-dependent pattern was also evident for GW (Fig. 2b). More strikingly, this pattern emerged through reduced cross-site variation rather than enhanced performance: while the ‘Magal’ cytoplasm exhibited strong site-to-site responsiveness (gaining up to + 13.4% in TGW from Mibhor to Yotveta in the case of RecipPop₂^50W_38^), the B1K-50-04 cytoplasm showed limited TGW variation between the two sites (varying by only up to 2.6% in the case of RecipPop₂^50W_38^, compared to the ‘Magal’ cytoplasm, which varied by 13.3% in the same population). This differential plasticity caused the cytoplasms to converge at Yotveta while diverging at Mibhor (Fig. 2b, left). In contrast, the RecipPop₄^Noga^ comparison showed minimal cytoplasmic differentiation at both sites (Fig. 2b, right), suggesting that this environmental buffering is a specific property of the B1K-50-04 cytoplasm.

### Morphological and photosynthetic traits underpin cytoplasmic divergence

**Fig. 3:**
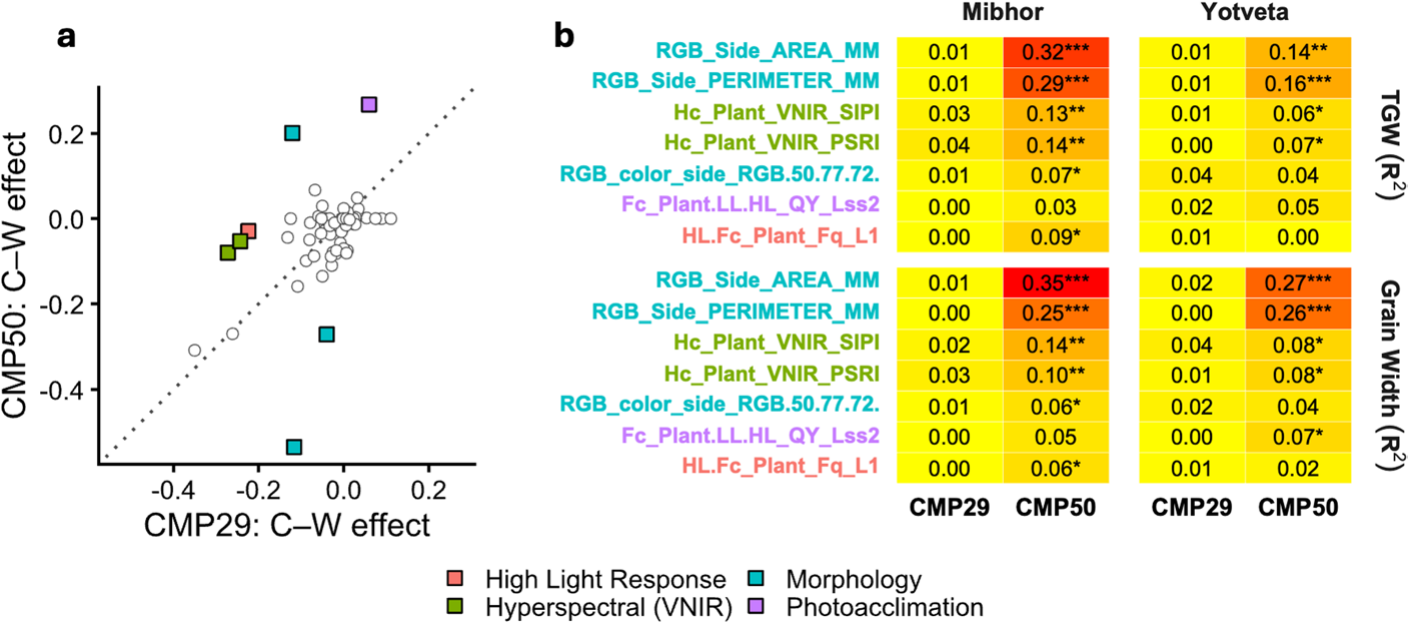
Phenomic signature of contrasting cytoplasmic effects. (**a**) Bivariate illustration of cytoplasmic effect sizes in the CMP29 and CMP50 families for 66 traits. Colored squares signify traits where divergence exceeds the threshold, with color indicating the trait modality. The dotted line marks the 1:1 expectation for identical cytoplasmic effects across CMP29 and CMP50. (**b**) Site-wise R^2^ values of correlations between divergent traits and TGW, grain width. The color of the trait names indicates the trait modality. ***p ≤ 0.001, **p ≤ 0.01, *p ≤ 0.05

Having established contrasting cytoplasmic effects on grain traits between CMP29 and CMP50, we assessed whether this contrast extends beyond grain traits using the PlantScreen phenomics platform (Abdelhakim et al. 2024; Tietze et al. 2025). We summarized the phenomics data as described in Methods, identifying seven highly divergent traits spanning four modalities (RGB morphology, hyperspectral VNIR reflectance, photoacclimation chlorophyll fluorescence, and high-light response; Fig. 3a). Of these, six exhibited concordant directional effects between CMP29 and CMP50, despite differing in magnitude; among them, morphological traits showed the strongest divergence, with plant area (RGB_Side_AREA_MM, divergence = 0.42) and perimeter (RGB_Side_PERIMETER_MM, divergence = 0.23) both displaying negative effects of the B1K-29-13 cytoplasm in CMP29 (weighted Cohen’s d = −0.12 and − 0.04, respectively) that were amplified with the B1K-50-04 cytoplasm in CMP50 (weighted d = −0.54 and − 0.27, respectively). Moderate divergence (0.19; Online Resource 9) was observed for canopy spectral reflectance indices, including plant senescence reflectance index (PSRI) and structure-insensitive pigment index (SIPI) (Peñuelas et al. 1995; Merzlyak et al. 1999), with both wild cytoplasms (B1K-29-13 and B1K-50-04) showing negative effects relative to ‘Noga’. Photoacclimation efficiency photosystem II quantum yield under actinic light steady state at 130 µmol m^−2^s^− 1^ (Fc_Plant.LL.HL_QY_Lss2) showed positive wild cytoplasm effects that diverged by 0.21 units between populations. Notably, one morphological color trait (RGB_color_side_RGB.50.77.72) exhibited opposite-direction effects, with the B1K-29-13 cytoplasm showing negative and the B1K-50-04 cytoplasm positive effects relative to ‘Noga’ (divergence = 0.32), representing the sole case of true antagonistic cytoplasmic action.

To evaluate whether divergent PlantScreen phenotypic traits mechanistically underpin the observed grain trait differences, we examined site-specific correlations between the seven divergent traits and both TGW and GW (Fig. 3b). Morphological traits (area and perimeter) displayed significant correlations with both TGW and GW in CMP50, with R^2^ values of 0.35 and 0.27 for GW at Mibhor and Yotveta, respectively, and 0.25 and 0.26 for TGW at the same sites.

For TGW, correlations were strongest at Mibhor, with R^2^ = 0.32 and R^2^ = 0.29 for RGB_Side_AREA_MM and RGB_Side_PERIMETER_MM, respectively, whereas at Yotveta, these values decreased to 0.14 and 0.16, respectively. Within CMP50, modest correlations were also observed between TGW and GW and the hyperspectral SIPI and PSRI traits at Mibhor (R² = 0.10–0.14; Fig. 3b), while the remaining traits showed weak (R^2^ < 0.09) or no consistent correlations across populations and environments.

### Environment-dependent cytonuclear epistasis reveals a chromosome 5 H hotspot

While the MRVP revealed no significant cytoplasm × environment interactions for most grain traits (Fig. 1c), reaction norm analysis suggested that cytoplasmic effects were often conditioned by one of the two field sites (Fig. 2b). This observation prompted us to investigate whether nuclear genetic variation might underlie these population- and environment-specific patterns of cytonuclear interaction. We performed a marker-based genetic analysis using a targeted panel of 35 KASP markers (Online Resource 11) positioned at previously identified MTAs, including cnQTL identified in the CMPP (Bodenheimer et al. 2025). This design allowed us to simultaneously validate earlier QTL signals in an independent genetic background while explicitly testing for marker × cytoplasm × environment (G × C × E) interactions. To expand our understanding of cytoplasmic effects beyond grain weight and morphology, we also incorporated NIRS measurements of grain compositional traits, including protein, fat, and ash, as a percentage of grain composition. NIRS concentrations were further combined with gravimetric yield data to produce derived mass traits representing total component mass per 1000 grains (Prot1000, Fat1000, Ash1000) and per plant (ProtP, FatP, AshP; see Methods).

**Fig. 4:**
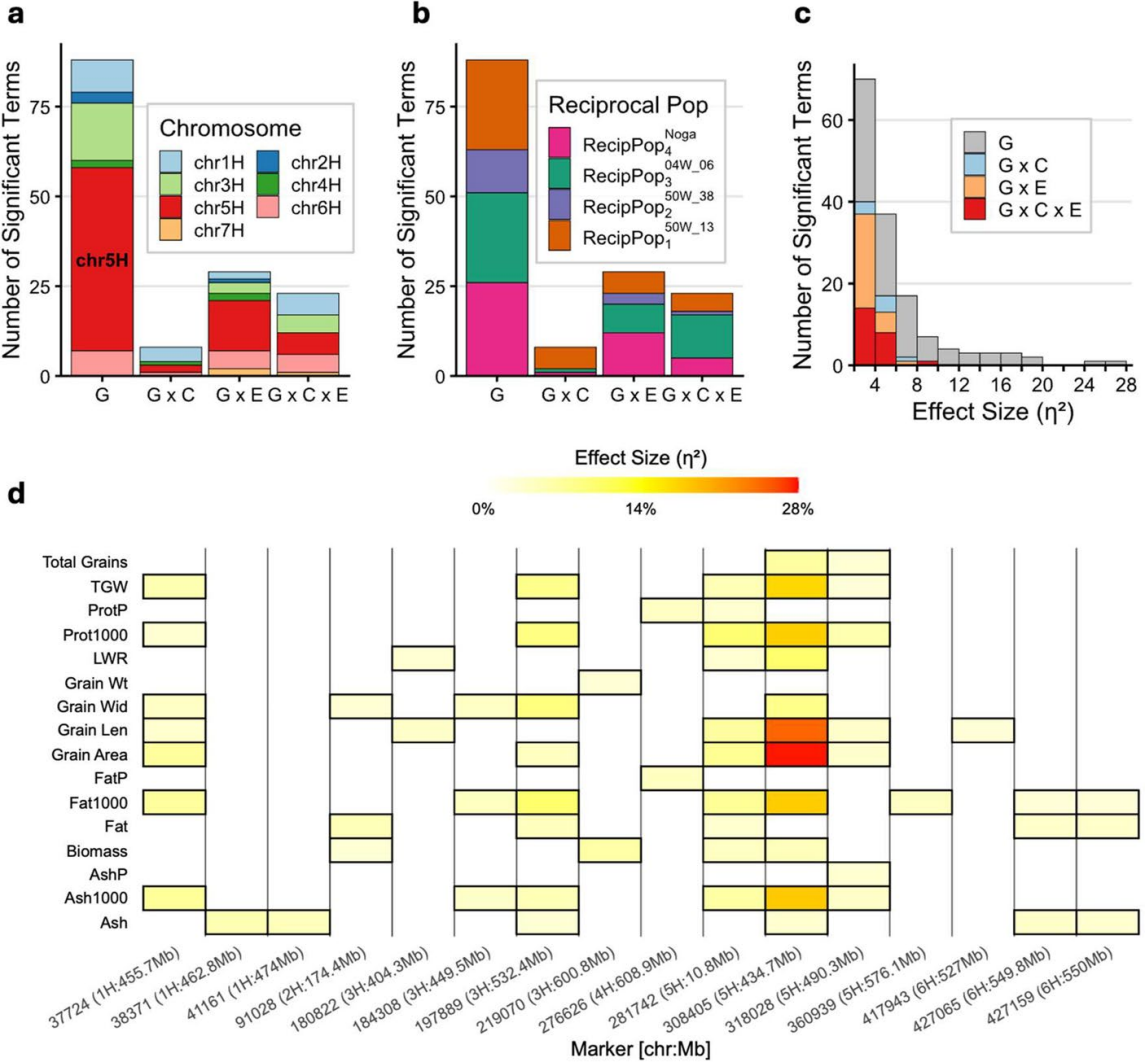
Three-way marker × cytoplasm × environment (G × C × E) analysis using a targeted 35-marker panel. (**a**, **b**) Term-wise distribution of significant terms, colored by chromosome (**a**) and MRVP population (**b)**. (**c**) Distribution of significant terms by their effect size (η²). (**d**) Maximum effect size by trait, for significant marker (G) effects across the MRVP populations

Across the four populations, we detected 149 significant terms spanning four interaction levels: 89 marker main effects (G), 8 marker-by-cytoplasm interactions (G × C), 29 marker-by-environment interactions (G × E), and 23 three-way interactions (G × C × E; Online Resource 12). Genomic distribution of these terms revealed that chromosome 5 H (Fig. 4a) harbored 74 of 149 effects (50%), including 52 of 89 (58%) marker main effects. Chromosomes 3 H and 1 H emerged as secondary hotspots, contributing 24 and 21 significant effects, respectively. The distribution of significant terms across the MRVP populations also revealed a population-specific pattern of nuclear–cytoplasmic interaction (Fig. 4b). While marker main effects were distributed evenly across populations, three-way interactions were markedly concentrated in RecipPop₃^04W_06^, which accounted for 12 of 23 (52%) G × C × E terms.

Effect sizes for marker main effects reached up to 27.8%, with 17 G-terms exceeding η² = 10% (Fig. 4c). This led us to examine a trait- and marker-resolved map of significant G-terms (Fig. 4 d), where JHI-Hv50k-2016–308405 emerged as the predominant driver of genetic variation across multiple grain traits. This marker, located on chromosome 5 H at 434.7 Mb, harbored the maximum observed G effect for eight traits spanning grain morphology and composition: GA (η² = 27.8%), GL (24.9%), Ash1000 (18.1%), Fat1000 (17.8%), Prot1000 (17.7%), TGW (17.0%), LWR (10.3%), and GPP (6.7%). The marker also showed significant G effects for GW (η² = 8.3%), although JHI-Hv50k-2016–197889 on chromosome 3 H narrowly exceeded it for this trait (9.1%; Online Resource 12). Together, these results indicate that the underlying locus influences grain size, weight, and number, as well as macronutrient composition on a per-grain basis.

**Fig. 5:**
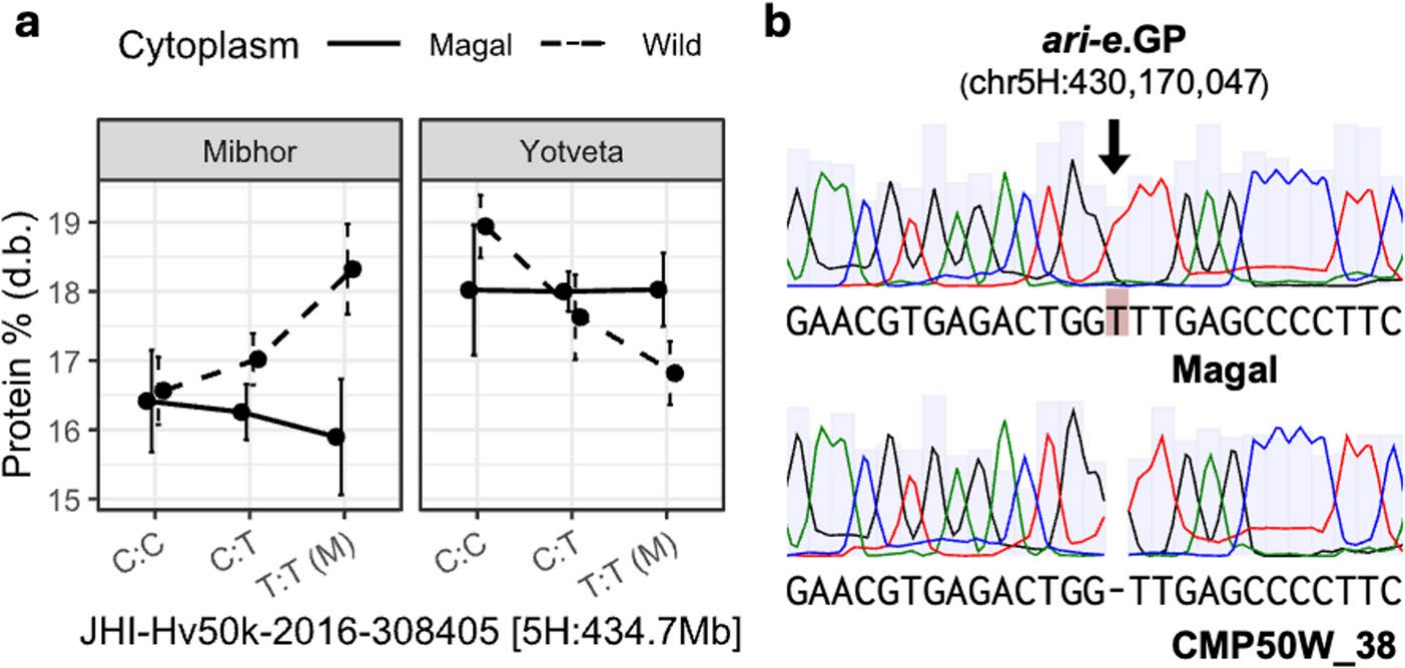
(**a**) Reaction norms for significant three-way interaction at the *ari-e* locus within the RecipPop_2_^50W_38^ population, with the wild cytoplasm representing B1K-50-04. The ‘Magal’ allele is referred to as (M); d.b., dry basis. (**b**) Sanger trace of the 1-bp *ari-e.GP* insertion in the mutant (‘Magal’) and wild-type (CMP50W_38) backgrounds

Asking whether this hotspot also contributes to cytonuclear epistasis, we found that 6 of 23 G × C × E interactions mapped to chromosome 5 H, with one involving JHI-Hv50k-2016–308405 specifically for protein % (dry basis) in RecipPop_2_^50W_38^ (η² = 3.6%; Fig. 5a): at Mibhor, the ‘Magal’-derived homozygote (T: T) increased protein content to 18.3 ± 0.65% (mean ± SE) in the B1K-50-04 cytoplasmic background, compared to 16.6 ± 0.49% for the alternative homozygote (C: C). In contrast, in the ‘Magal’ cytoplasmic background, the same allele showed no advantage, with trait values comparable to the founder allele (15.9 ± 0.84% vs. 16.4 ± 0.73%). The pattern shifted at Yotveta, where the ‘Magal’-derived allele increased protein content in the ‘Magal’ cytoplasmic background relative to its performance in the B1K-50-04 background (18.0 ± 0.53% vs. 16.8 ± 0.46%). Beyond chromosome 5 H, three-way interactions were particularly concentrated on chromosomes 1 H (6 G × C × E terms) and 3 H (5 G × C × E terms), where they involved both morphological and compositional traits. The six derived mass traits contributed 6 of 23 (26%) G × C × E interactions overall, including terms for Prot1000, FatP, ProtP, and AshP, indicating that cytonuclear epistasis shapes not only grain concentration but also the total mass of grain components.

The accumulation of both marker main effects and G × C × E interactions on chromosome 5 H, particularly clustered near 434.7 Mb, encouraged us to investigate the underlying genetic basis of this region. Cv. Magal contains germplasm from the Australian Hindmarsh variety (see Methods), which carries the *ari-e.GP* semi-dwarfing allele (Liu et al. 2014; Fox and Lance 2020). This allele was previously mapped to the *HvDep1* gene, a Gγ subunit of a heterotrimeric G‑protein complex shown to influence grain size and plant architecture (Wendt et al. 2016). *HvDep1* is located on chromosome 5 H at 430.2 Mb, positioning it within 4.5 Mb of JHI-Hv50k-2016–308405. This close physical proximity, combined with the large phenotypic effects and multitrait pleiotropy described previously (Dockter and Hansson 2015), identified *ari-e* as a strong candidate for the observed hotspot. In fact, Sanger sequencing confirmed that ‘Magal’ does carry the *ari-e.GP* insertion while CMP50W_38, representative of the remaining MRVP donors, features the wild-type allele (Fig. 5), establishing segregation at this locus in the MRVP, with the T allele at marker JHI-Hv50k-2016–308405 corresponding to *ari-e.GP* and the C allele to the wild type. Since the MRVP donors (CMP50W_13, CMP50W_38, CMP04W_06) also carry the ‘Noga’-derived C allele at this marker (Bodenheimer et al. 2025), this G × C × E interaction reflects cytoplasm-dependent effects on allelic variation between two cultivated backgrounds.

## Discussion

### A portable, buffering wild cytoplasm

Our results collectively support the notion that cytoplasmic variation is not merely a background effect but a distinct, heritable source of phenotypic variation in barley. We validated the effects of the wild B1K-50-04 cytoplasm, which consistently provided a TGW advantage across different nuclear backgrounds and environments. Crucially, this study demonstrates the portability of these cytoplasmic effects. The transition from the original CMPP donors (‘Noga’) to the elite ‘Magal’ background (MRVP) did not attenuate the cytoplasmic benefit; rather, the B1K-50-04 cytoplasm maintained, or even amplified, its positive effect on TGW (Fig. 1c). This suggests that favorable wild cytoplasms can be introduced into modern lines with an overall reduced risk of deleterious effects that are often associated with wild nuclear introgressions. Notably, in the current study, the phenotypic contrast between carriers of two cultivated alleles (at JHI-Hv50k-2016–308405 on chromosome 5 H) was conditioned by the presence of the B1K-50-04 cytoplasm (Fig. 5a). These results highlight the potential of this cytoplasmic swapping and the resulting epistatic interactions to reveal allelic effects within the cultivated gene pool that remain hidden in a single cytoplasmic background.

A striking feature of the wild cytoplasm is its reduced cross-environment responsiveness. While the cultivated ‘Magal’ cytoplasm exhibited high plasticity, reacting strongly to the environmental shift between Mibhor and Yotveta (Fig. 2b), the B1K-50-04 cytoplasm buffered this response, maintaining consistent grain characteristics across contrasting conditions. Although our two-environment design does not permit formal stability analysis (e.g., Shukla’s variance or Finlay–Wilkinson regression), the pattern observed here is consistent with the improved TGW stability previously documented for the B1K-50-04 cytoplasm within the CMP50 subfamily evaluated across four environments (Bodenheimer et al. 2025), where Shukla’s stability variance dropped from 8.89 in the ‘Noga’ cytoplasmic portion of the subfamily to 1.04 in the B1K-50-04 portion. This builds on recent findings linking specific plastidial genes, such as *RpoC1*, to thermal plasticity (Tiwari et al. 2024), suggesting that the B1K-50-04 cytoplasm may harbor variants that optimize developmental and biochemical homeostasis under stress.

### Pinpointing the causality underlying cytonuclear effects

While we previously highlighted *RpoC1* as a candidate plastid locus for the cytoplasmic side of the cytonuclear interactions (Tiwari et al. 2024), and here identify *HvDep1* for the nuclear side (see further on), definitive attribution requires precise genetic manipulation. Current gene-editing technologies (e.g., TALENs, CRISPR-Cas9) are well-established for nuclear genes, but targeted manipulation of organellar genomes remains technically demanding (Forner and Bock 2025). Until organellar editing becomes routine in barley, developing reciprocal near-isogenic lines targeting the *ari-e* locus in diverse cytoplasmic backgrounds is essential to fully dissecting the physiological mechanisms driving this interaction. Nevertheless, this finding serves as both a cautionary and opportunistic tale for breeding: the performance of major agronomic genes such as *ari-e* can be contingent on variation in the cytoplasmic “second genome.”

### Methodological considerations and breeding integration

The detection of cytoplasmic and cytonuclear effects relies heavily on experimental design. While the use of reciprocal F_2_ populations in the MRVP allowed for an immediate assessment of cytoplasmic effects in a segregating nuclear background, this approach has limitations with respect to plot-level inference of grain yield. The high phenotypic noise inherent to single-plant measurements in F_2_ populations (as evidenced by the high CV for GPS) masks subtle cytonuclear interactions that might influence total grain yield rather than just grain weight components. Accordingly, we interpret the MRVP primarily in terms of grain weight and size components and their cross-site consistency, rather than as a definitive test of yield gains. Moreover, increased grain weight does not always translate into yield gains. Recent multiyear field trials of *TaGW2* near-isogenic lines in wheat demonstrated that despite 20% increases in TGW, overall grain yield remained unchanged or was slightly reduced due to compensatory trade-offs with grain number (Simmonds et al. 2025), underscoring the importance of evaluating cytoplasmic effects on total grain yield rather than focusing solely on TGW. To address this, future strategies should prioritize the development of reciprocal recombinant inbred lines that would allow for replicated plot trials, reduce environmental error, and provide the necessary statistical power to detect smaller-effect cnQTL that likely segregate alongside major drivers such as *ari-e*.

Integrating cytoplasmic variation into genomic selection cycles is an important next step. Analyzing the full CMPP, we previously developed a genomic prediction strategy incorporating interactions between significant MTAs and population-structure variables (subfamily and cytoplasm) (Bodenheimer et al. 2025). Despite using a markedly smaller number of predictors, thereby reducing the computational time, these models achieved predictive accuracies comparable to or higher than those obtained with the complete 6,679-SNP dataset. Breeding programs traditionally treat the cytoplasm as a fixed factor. However, here as well, our data indicate that the cytoplasm explains a significant portion of the variance (η^2^ up to 9.5% for TGW). In prediction models, treating the cytoplasm as an independent genetic term could improve prediction accuracy, particularly for traits showing G × E interactions. Building on the formal stability evidence from the CMPP (Bodenheimer et al. 2025) where we established that specific wild cytoplasms confer environmental buffering, “cytoplasmic selection” could be deployed to breed varieties that are specifically adapted to variable climate scenarios, effectively harnessing this maternal component of adaptation that is often ignored in standard genomic selection pipelines.

On the flip side, exploiting cytoplasmic variation adds complexity to breeding pipelines. Reciprocal crossing schemes double the crossing effort, and the cytonuclear epistasis documented here, where the effect of a major nuclear locus (*ari-e*) depends on the cytoplasmic background, means that allelic effects estimated in one cytoplasm cannot be assumed to transfer to another. Breeders will need to weigh this added dimensionality against the grain-trait gains demonstrated here.

#### Phenomics: linking vegetative signatures to grain yield components

The deployment of high-throughput phenomics might provide a mechanistic bridge between early vegetative growth and final grain traits. We screened the PlantScreen phenomics data for vegetative traits whose cytoplasmic effect (wild vs. ‘Noga’) differed most between the CMP29 and CMP50 families, i.e., traits capturing the contrasting grain-trait signatures of the B1K-29-13 and B1K-50-04 cytoplasms. This yielded a subset of morphological and canopy spectral reflectance indices whose values correlated with GW and TGW, with the strength of those correlations varying by population and site (Fig. 3b). Together, these associations suggest that the cytoplasmic grain-trait advantage is set up early in vegetative development, possibly through differences in pigment allocation and growth-rate dynamics. Moreover, the correlation between vegetative plant area (as manifested by RGB_Side_AREA_MM, which represents a RGB-based biomass proxy, Fig. 3) and final GW suggests that either the cytoplasmic advantage is established early in development, or cytoplasmic effects can also be observed for early developmental traits that may relate directly or indirectly to GW. This could challenge the assumption that variation in grain filling is largely driven by differences in late-stage assimilation supply (Waters et al. 2009). Instead, the data support a model in which cytoplasmic variation influences early-stage photosynthetic efficiency or carbon partitioning, establishing a “biomass reserve” that supports superior grain filling later in the season. These non-destructive, early-stage metrics offer a scalable selection tool for breeders to screen for favorable combinations before harvest (Tietze et al. 2025), although whether predictions can be transferred from pot experiments to a field set-up remains an open question. It should be noted that these early predictors, or out-of-field predictions (on a moving field in a greenhouse as in this study), may be less valid for estimating the total yield of plants grown under high temperature. We have previously shown that some QTL underlying thermal plasticity of the circadian clock, as determined by the SensyPAM platform in controlled environments, are pleiotropic for increased biomass and reduced grain yield under field experiments (Prusty et al. 2021), presumably due to a change in the source–sink relationship. These observations suggest that while phenomics under optimal conditions could provide early-stage predictions for later reproductive phenotypes under non-stressed conditions (current study), these predictions may fail under conditions of abiotic stress in the field.

### Deciphering the *ari-e* cytonuclear hotspot

One of the most significant findings of this study is the identification of a G × C × E interaction hotspot on chromosome 5 H, colocalizing with the *ari-e* locus. By sequencing the *HvDep1* gene, we confirmed that segregation of the semi-dwarfing *ari-e.GP* allele vs. the wild-type allele drives this interaction. The *ari-e.GP* allele is widely deployed for its contribution to lodging resistance and harvest index (Dockter and Hansson 2015). However, our results show that its effect on grain quality (protein content) is not autonomous but depends on the cytoplasmic background and the environment. To our knowledge, this effect of ari-e (Gϒ-subunit, (Wendt et al. 2016) mutant haplotypes on protein content has not been observed previously, whereas mutations in a different (Gα) subunit do not have an effect on grain quality (Braumann et al. 2017). Moreover, our observation that the *ari-e.GP* allele only significantly increased protein content in the presence of the B1K-50-04 cytoplasm at Mibhor (Fig. 5) suggests specific cytonuclear epistasis. HvDep1 encodes the γ-subunit of a heterotrimeric G protein involved in transmembrane signaling (Wendt et al. 2016). It is plausible that the altered signaling cascade triggered by ari-e.GP requires a specific bioenergetic state, which B1K-50-04 cytoplasms may help establish, thereby optimizing nitrogen remobilization into the grain. This cytoplasm-dependent behavior of a major dwarfing gene parallels recent findings in wheat, where the phenotypic effects of TaGW2 grain-size mutations were differentially modulated by the semi-dwarfing alleles RHT-B1b and RHT-D1b (Simmonds et al. 2025). Together, these results indicate that the breeding value of individual loci cannot be assessed in isolation from their nuclear genetic background or, as shown here, from their cytoplasmic context. Future studies should address the spatiotemporal changes in the relevant molecular pathways underlying grain development to elucidate the mechanisms underlying the cytoplasm-conditioned effects of *HvDep1*.

## Supplementary Information

Below is the link to the electronic supplementary material.

Supplementary Material 1 (ZIP 5.74 MB)

Supplementary Material 2 (R 39.3 KB)

Supplementary Material 3 (PDF 6.25 MB)

## Data Availability

The raw data (Online Resources 3 and 4), as well as the statistical output (Online Resources 8, 9, and 12) are available as part of the Electronic Supplementary Material of this article. An in-house custom NIRS calibration script will be provided as supplemental material during the review process and made available in a public GitHub repository with digital object identifier generated via Zenodo at the time of publication.
