# Supplementary material for: Wild barley cytoplasms reduce grain weight plasticity, with environment-dependent cytonuclear epistasis at the ari-e locus
Source: Mol Breed. 2026 May 19;46(6):48. doi: 10.1007/s11032-026-01673-6 (PMC13187085; doi:10.1007/s11032-026-01673-6)
Supplement: Supplementary file 1 — Supplementary Material 1 (ZIP 5.74 MB) [file 11032_2026_1673_MOESM1_ESM.zip › Supplementary Material/ESM_Legends.docx]

# Electronic Supplementary Material Legends

## Online Resource 1

**Composition and experimental design of the CMPP Validation Panel (CMPPV) and the Magal Reciprocal Validation Panel (MRVP).** Each panel is defined by the populations tested, reciprocal parental combinations, generation type and segregation status, sample sizes in Mibhor (M) and Yotveta (Y), environments and experimental designs, plot structure and harvest unit, and recorded field traits (GA, Grain Area; GL, Grain Length; GW, Grain Width; GPS, Grains per Spike; LWR, Length-Width Ratio; TGW, Thousand Grain Weight; BM, Biomass; GWP, Grain Weight per Plant; GPP, Grains per Plant. For both panels, trials were planted side-by-side in 2023 and harvested in 2024.

## Online Resource 2

**Comparison of grain dimensions for manually calculated, vs. auto-calculated values using the Marvin 6 software.**

## Online Resource 3

**Raw phenotypic data for the CMPP Validation Panel (CMPPV).** Variables represent panel identifier, population label, line identifier, environment, cytoplasm descriptors, and recorded phenotypes.

## Online Resource 4

**Raw phenotypic data for the Magal Reciprocal Validation Panel (MRVP).** Variables represent panel identifier, population label, environment, plant identifier, cross designation, and recorded and derived phenotypes.

## Online Resource 5

**Schematic overview of the PlantScreen phenomic workflow.**

## Online Resource 6

**Distribution of Global and Neighborhood H values for MRVP grain samples**. Scanned with <25 g (a) and with >=25 g of sample mass (b).

## Online Resource 7

**Comparison of prediction performance (R^2^) against wet-chemistry reference values for the final custom NIRS calibration.** (a) Correlation with manufacturer calibration, (b) correlation with custom calibration and the pre-existing Cereals/Grains manufacturer calibration, reported as coefficient of determination.

## Online Resource 8

**Eta squared effect sizes from cytonuclear interaction analysis.** ANOVA results for all population-trait combinations from CMPPV and MRVP panels. Variables represent panel, phenotype descriptors, measurement unit, model term label, p-value, eta squared (η²), and 95% confidence interval bounds.

## Online Resource 9

**Summary of divergence statistics for phenomic comparison between CMP29 and CMP50.** Variables represent trait name, mean Cohen's d for CMP29, mean Cohen's d for CMP50, weighted Cohen's d for CMP29, weighted Cohen's d for CMP50, number of significant measurement days per population, divergence, effect direction, phenomics modality classification, and significance flag.

## Online Resource 10

**Trait divergence threshold.** Strip plot showing the absolute difference in weighted Cohen's d effect sizes between CMP50 and CMP29 for 66 PlantScreen phenomics traits. Dashed red line indicates the 90th percentile threshold used to classify traits as significantly divergent between the two populations.

## Online Resource 11

**KASP marker map for MRVP genotyping.** Genomic positions of 35 Kompetitive Allele Specific PCR (KASP) markers used to genotype the Magal Reciprocal Validation Panel. Positions refer to MorexV3.

## Online Resource 12

**Eta squared effect sizes from G × C × E analysis.** Variables represent panel, population label, phenotype code and abbreviation, official trait name, unit, marker identifier, chromosome identifiers, physical position, term, sample size, p-value, lower 95% CI bound, and eta squared (η²).

## Online Resource 13

**Daily mean air temperature at trial sites.** Mibhor was plotted against Yotveta during the trial period (December 2023 - May 2024). Each point represents one trial day (n = 155), with color coding representing chronological proximity to sowing (green) or harvesting (yellow). The dashed diagonal line indicates the 1:1 relationship.

## Online Resource 14

**Juxtaposition of 10 grains from parents used for the Magal Reciprocal Validation Panel (MRVP).** Scale bar equals 1 cm.

## Online Resource 15

**Summary statistics for the MRVP population and founder lines.**

## Online Resource 16

**Pairwise correlation matrix for grain traits in the CMPPV population.**

## Online Resource 17

**Site-level summary statistics for grain traits in the CMPPV.** SD, Standard Deviation; CV, Coefficient of Variation.
