# Supplementary figures and images for: Wild barley cytoplasms reduce grain weight plasticity, with environment-dependent cytonuclear epistasis at the ari-e locus
Source: Mol Breed. 2026 May 19;46(6):48. doi: 10.1007/s11032-026-01673-6 (PMC13187085; doi:10.1007/s11032-026-01673-6)

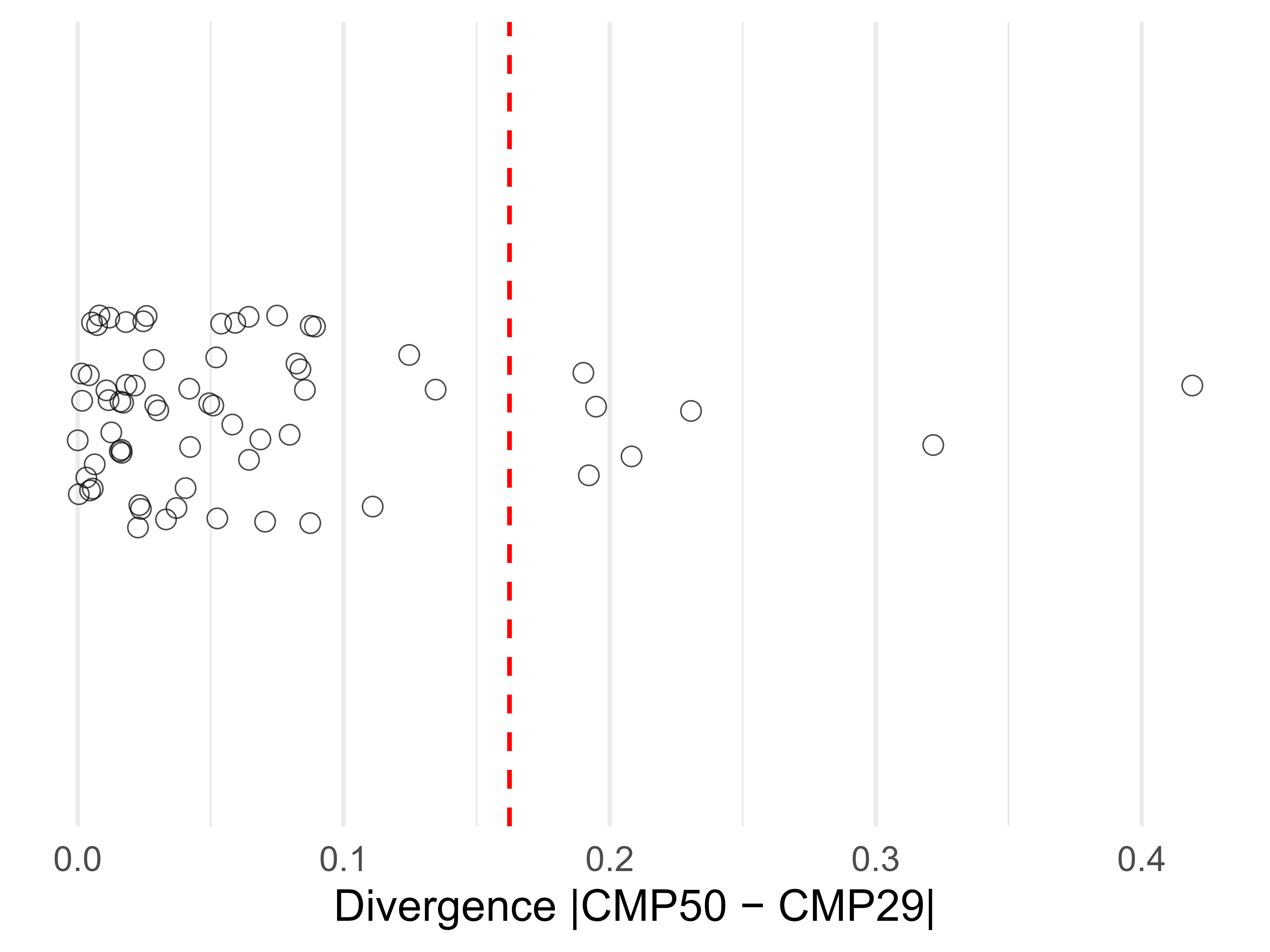

Supplement: Supplementary file 1 — Supplementary Material 1 (ZIP 5.74 MB) [file 11032_2026_1673_MOESM1_ESM.zip › Supplementary Material/ESM_10.png]

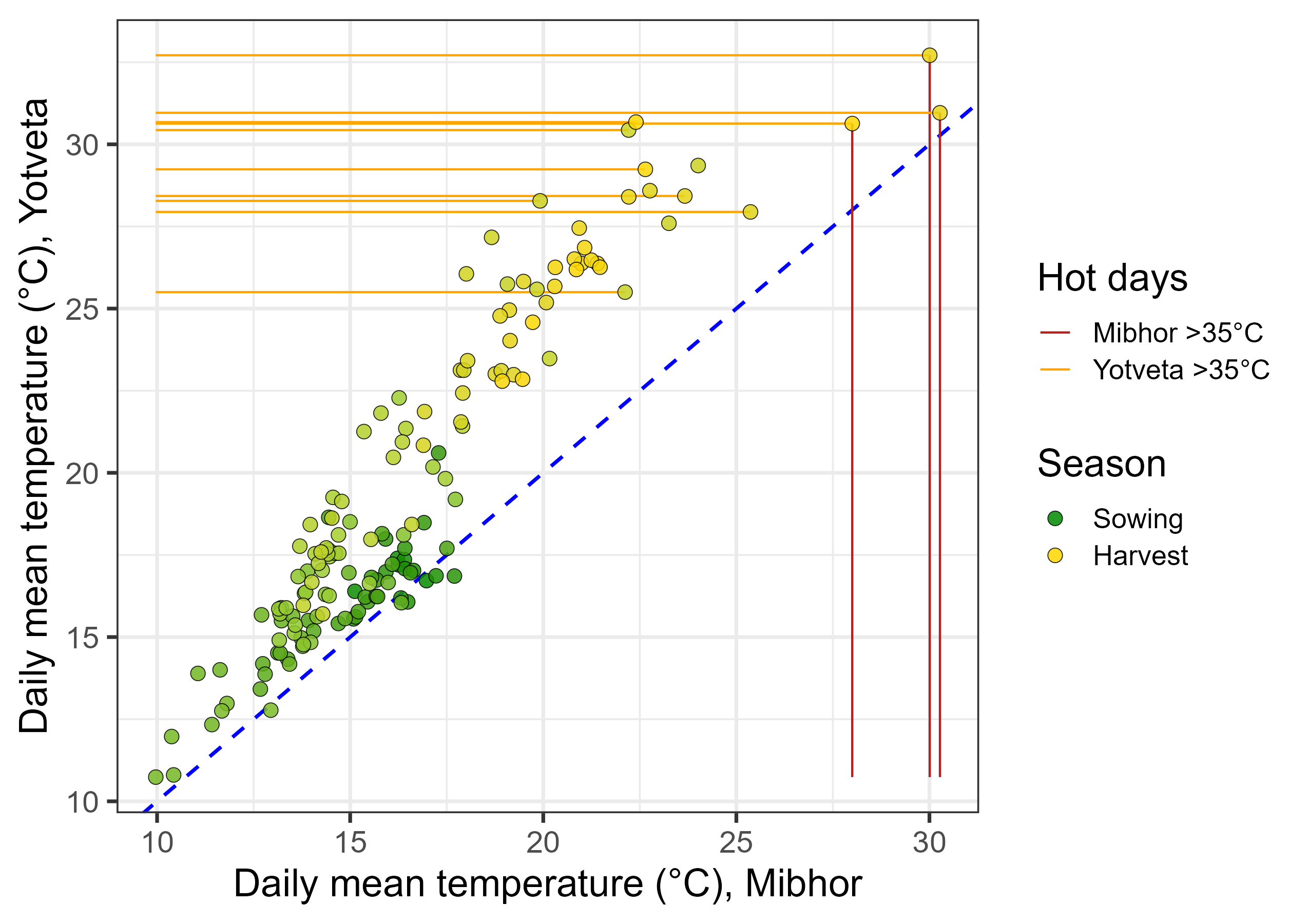

Supplement: Supplementary file 1 — Supplementary Material 1 (ZIP 5.74 MB) [file 11032_2026_1673_MOESM1_ESM.zip › Supplementary Material/ESM_13.png]

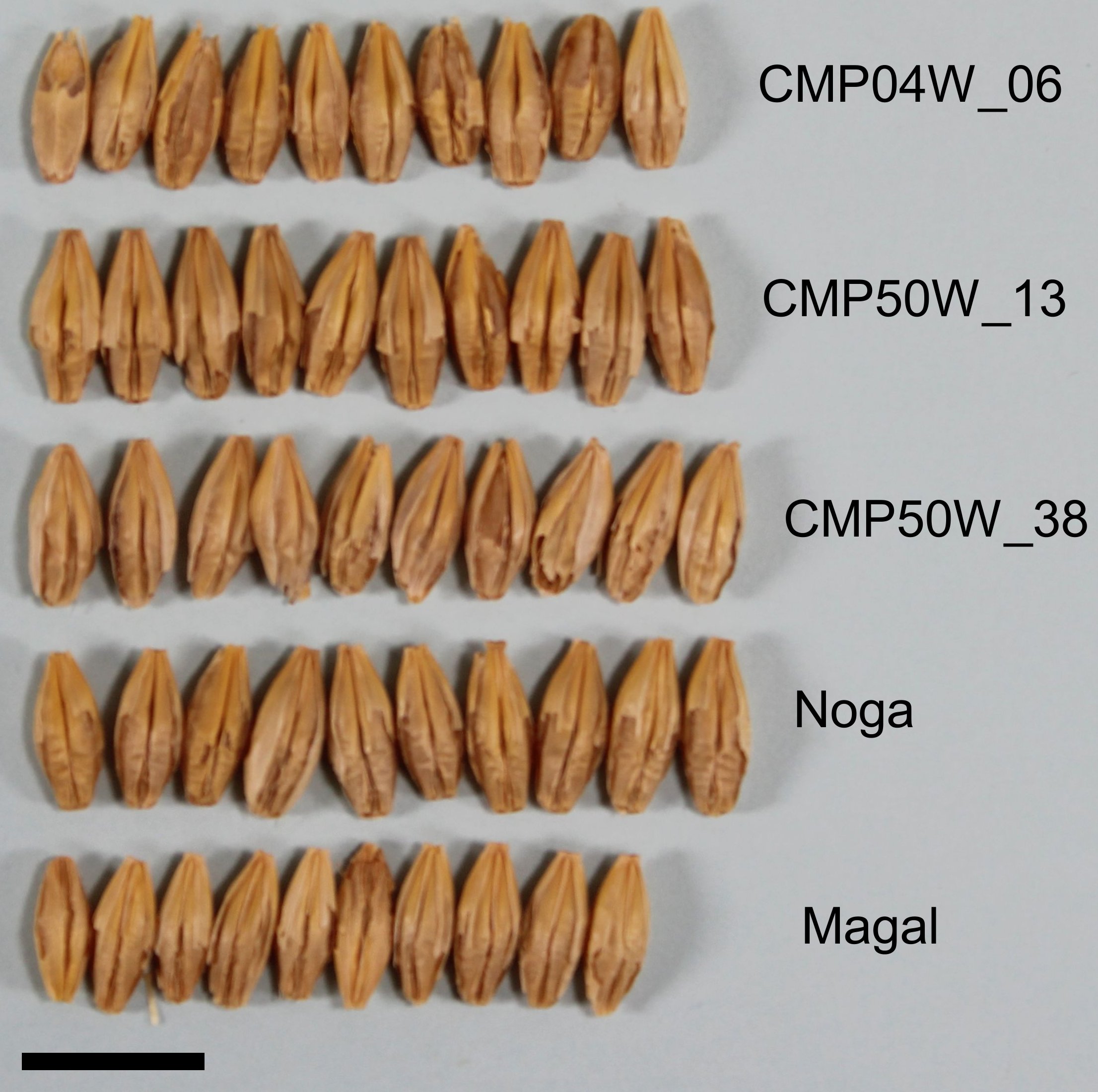

Supplement: Supplementary file 1 — Supplementary Material 1 (ZIP 5.74 MB) [file 11032_2026_1673_MOESM1_ESM.zip › Supplementary Material/ESM_14.jpg]

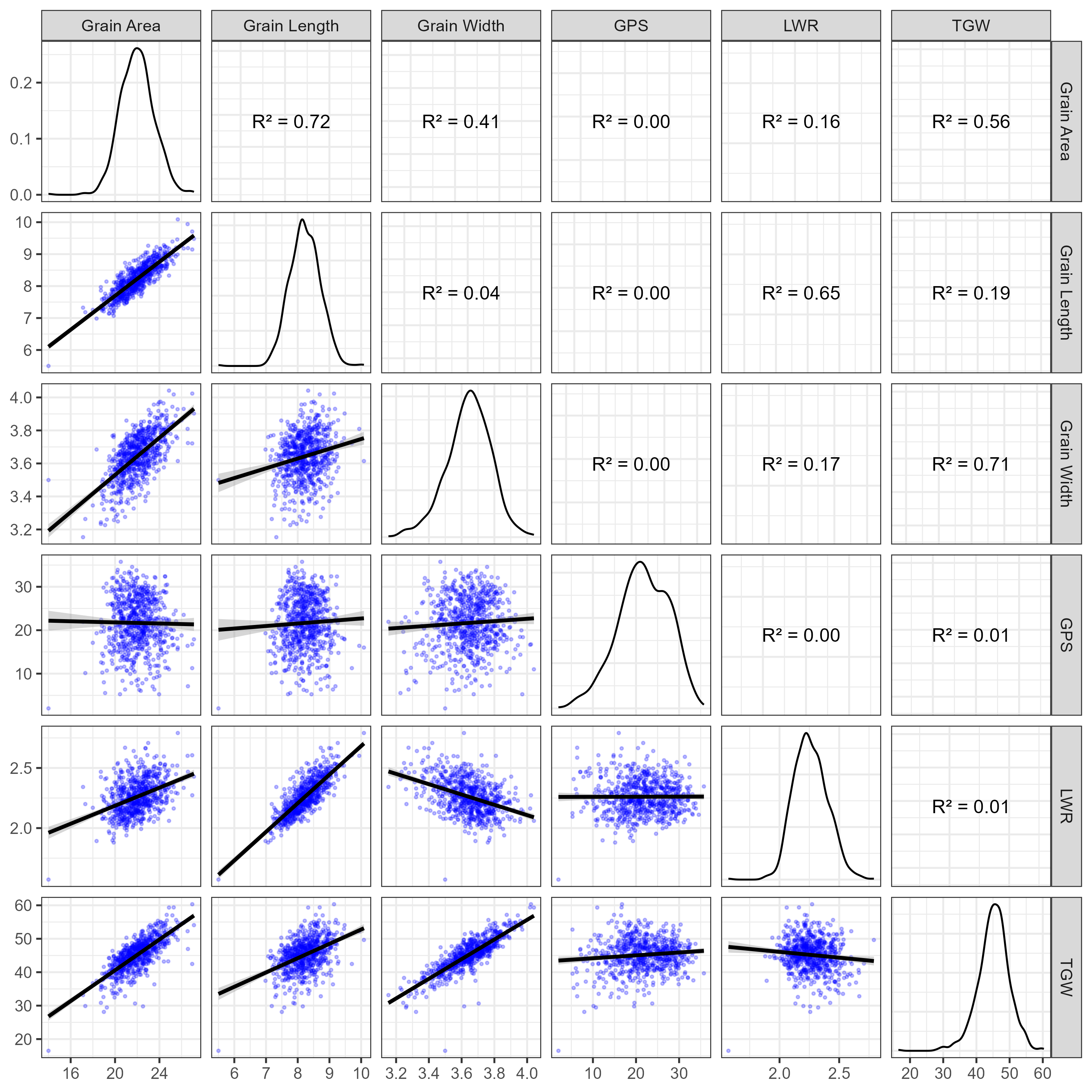

Supplement: Supplementary file 1 — Supplementary Material 1 (ZIP 5.74 MB) [file 11032_2026_1673_MOESM1_ESM.zip › Supplementary Material/ESM_16.png]

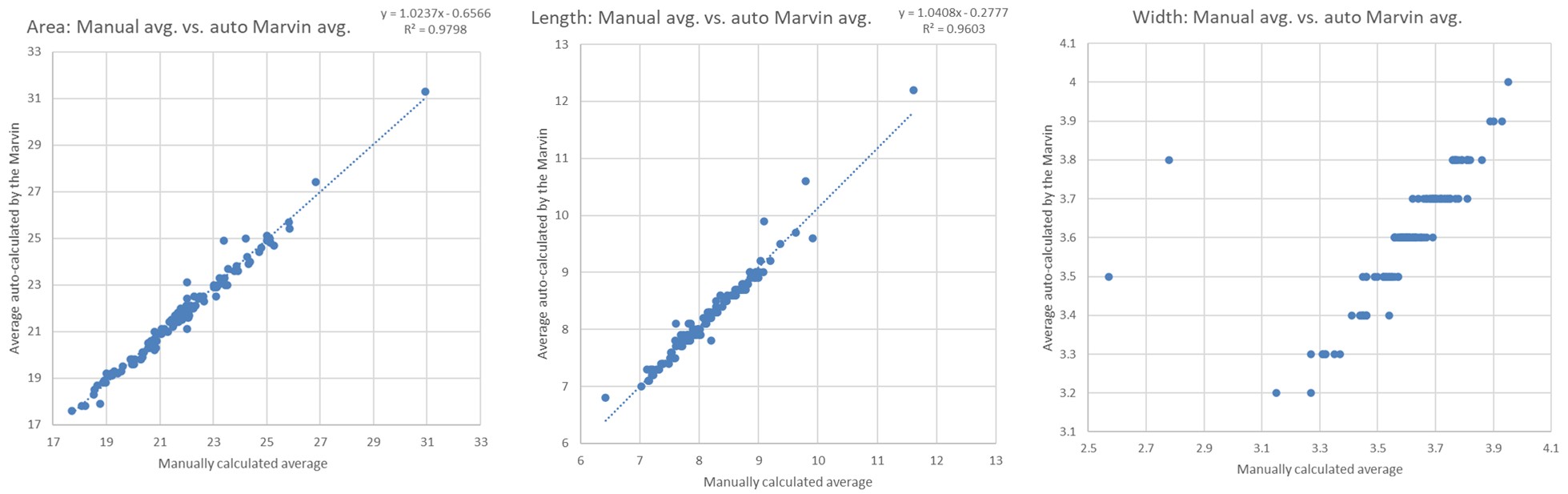

Supplement: Supplementary file 1 — Supplementary Material 1 (ZIP 5.74 MB) [file 11032_2026_1673_MOESM1_ESM.zip › Supplementary Material/ESM_2.jpg]

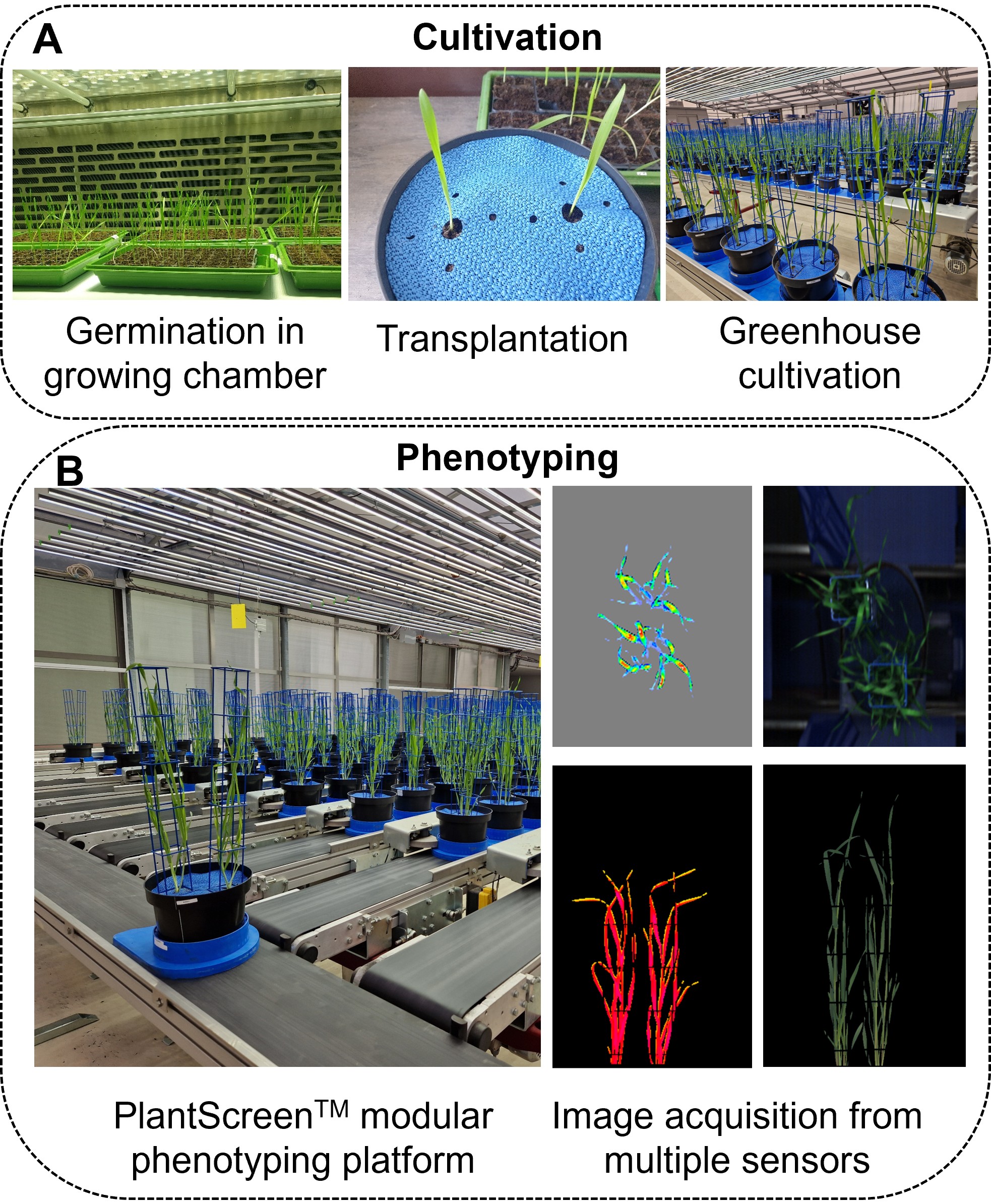

Supplement: Supplementary file 1 — Supplementary Material 1 (ZIP 5.74 MB) [file 11032_2026_1673_MOESM1_ESM.zip › Supplementary Material/ESM_5.jpg]

**a**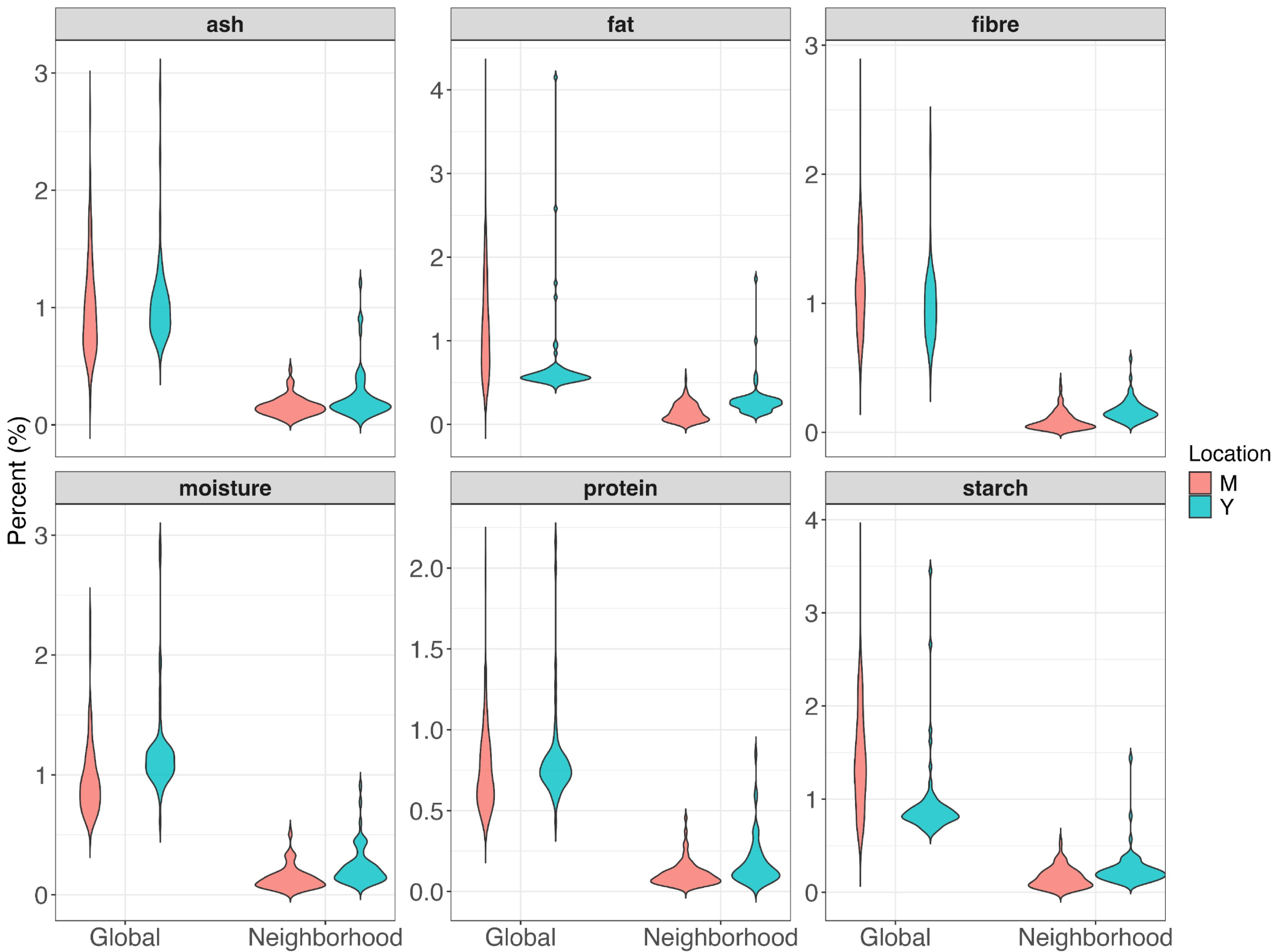**b**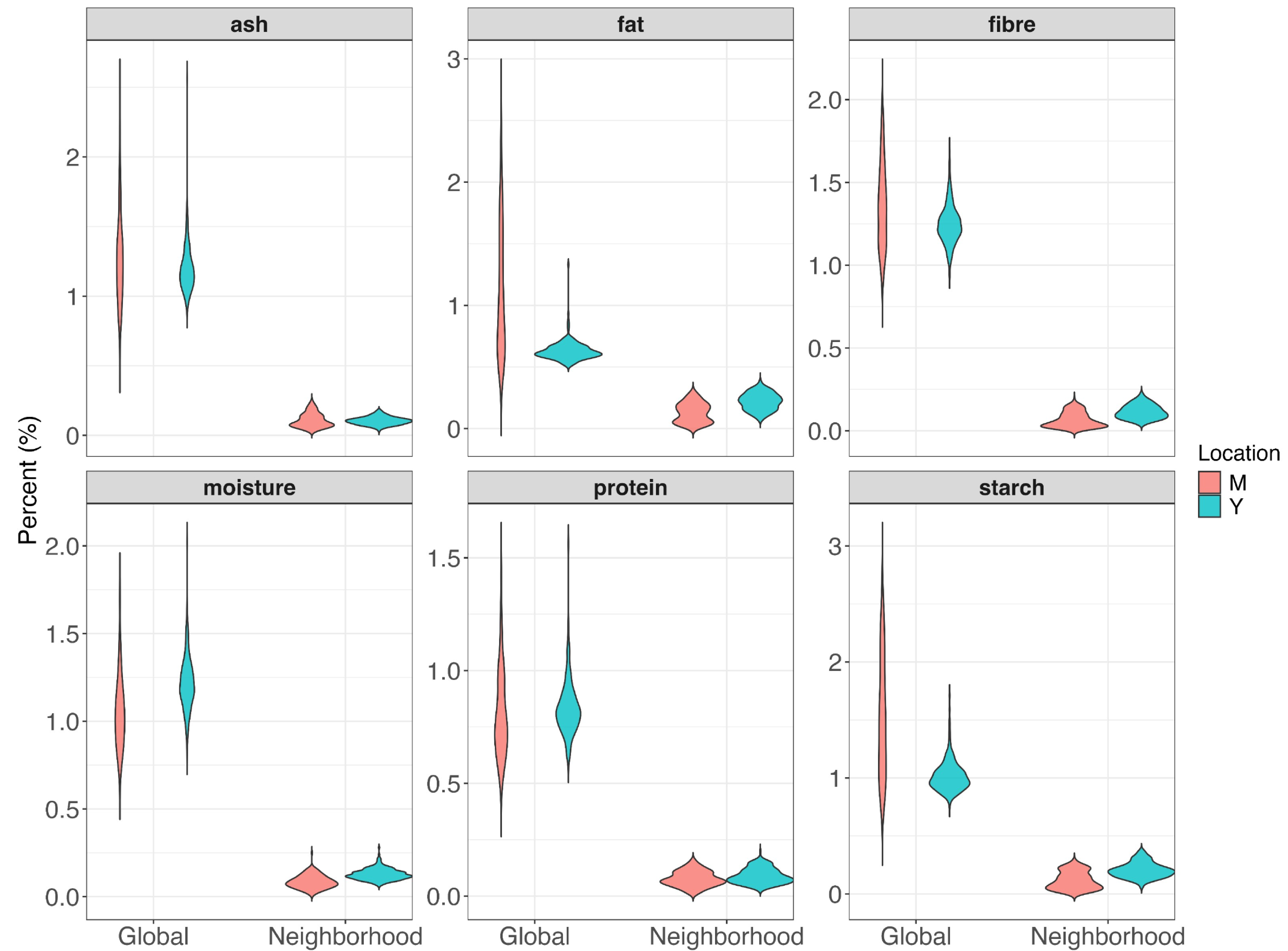

Supplement: Supplementary file 1 — Supplementary Material 1 (ZIP 5.74 MB) [file 11032_2026_1673_MOESM1_ESM.zip › Supplementary Material/ESM_6.pdf]

**a**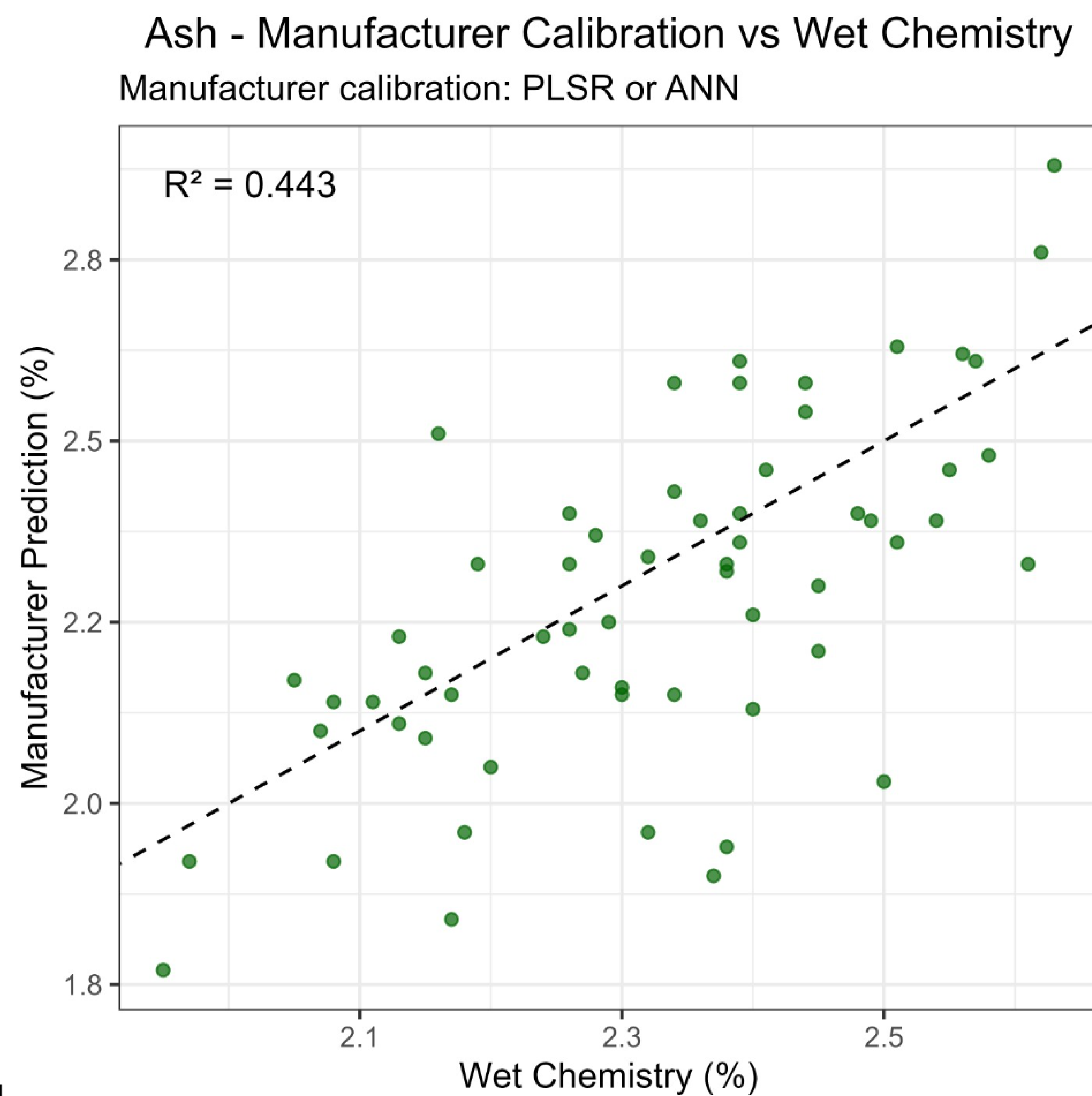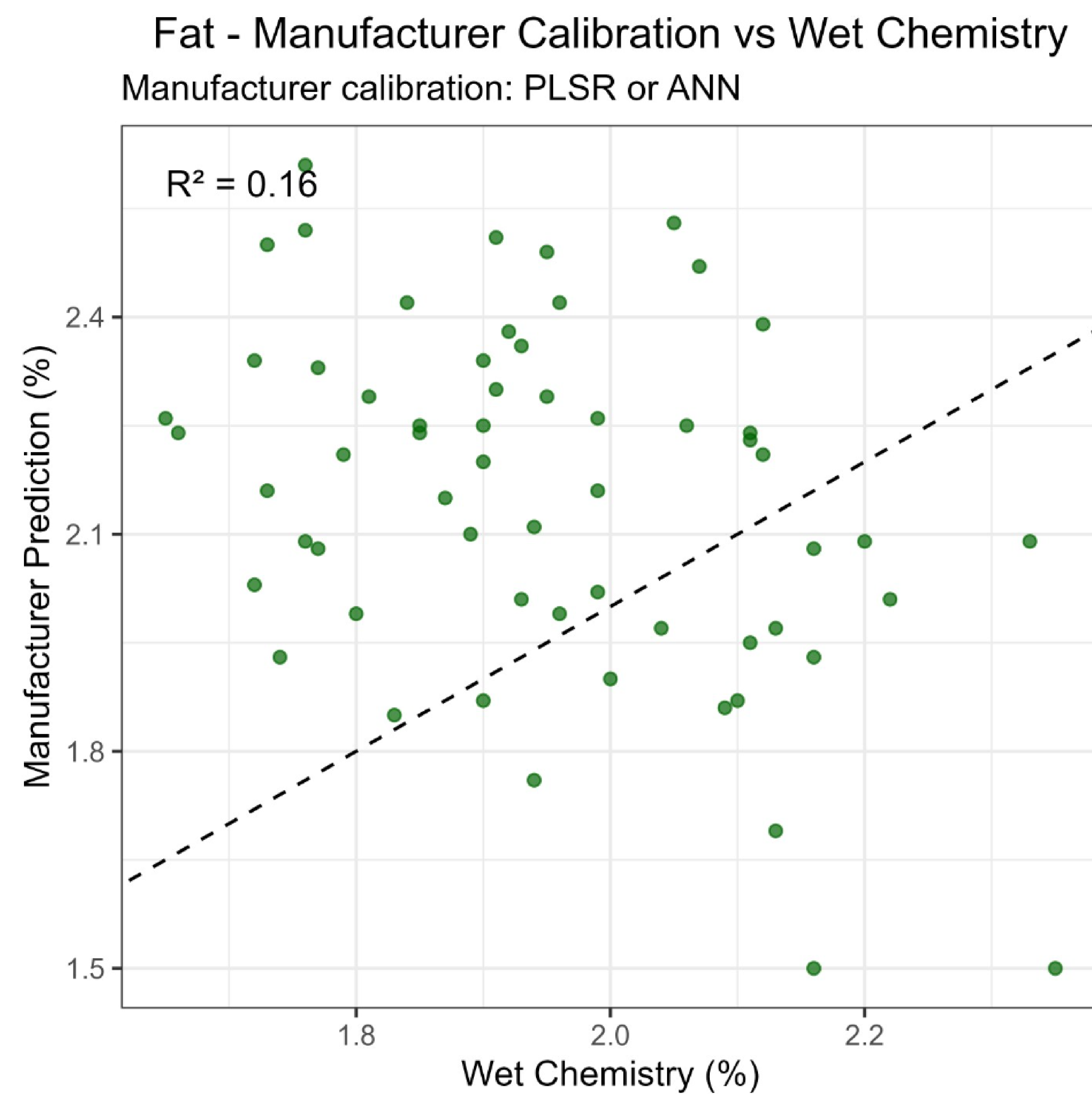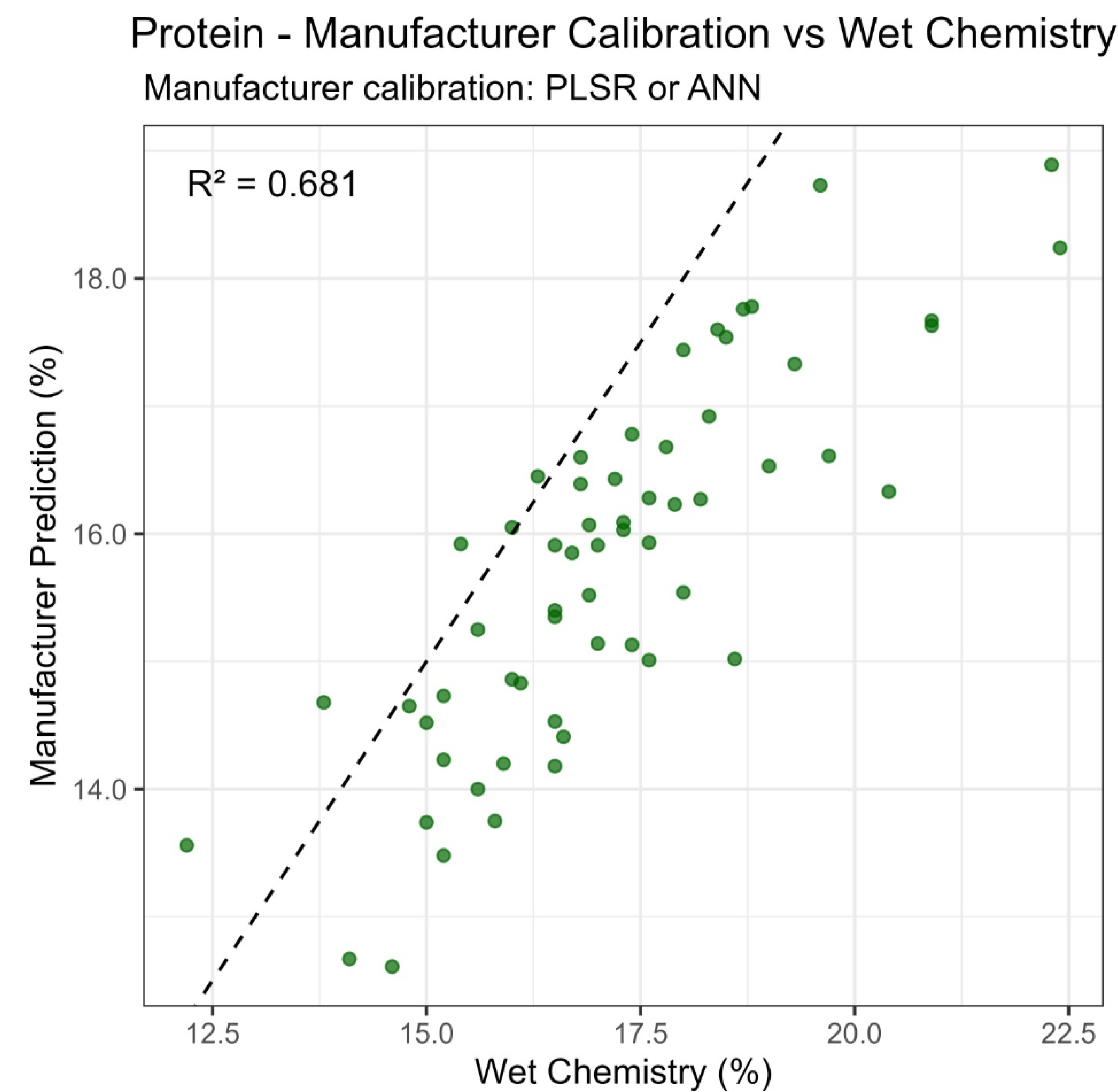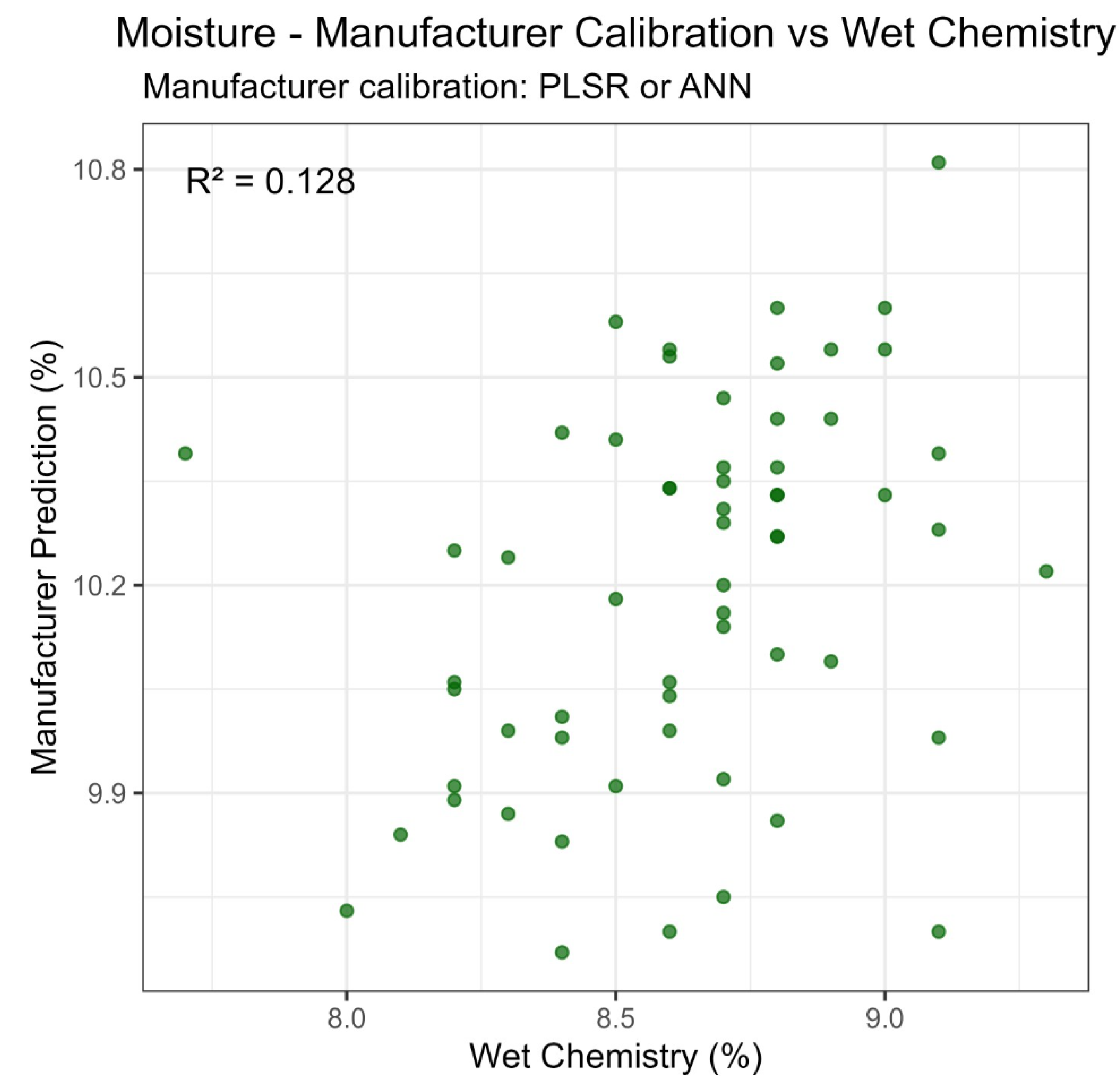**b**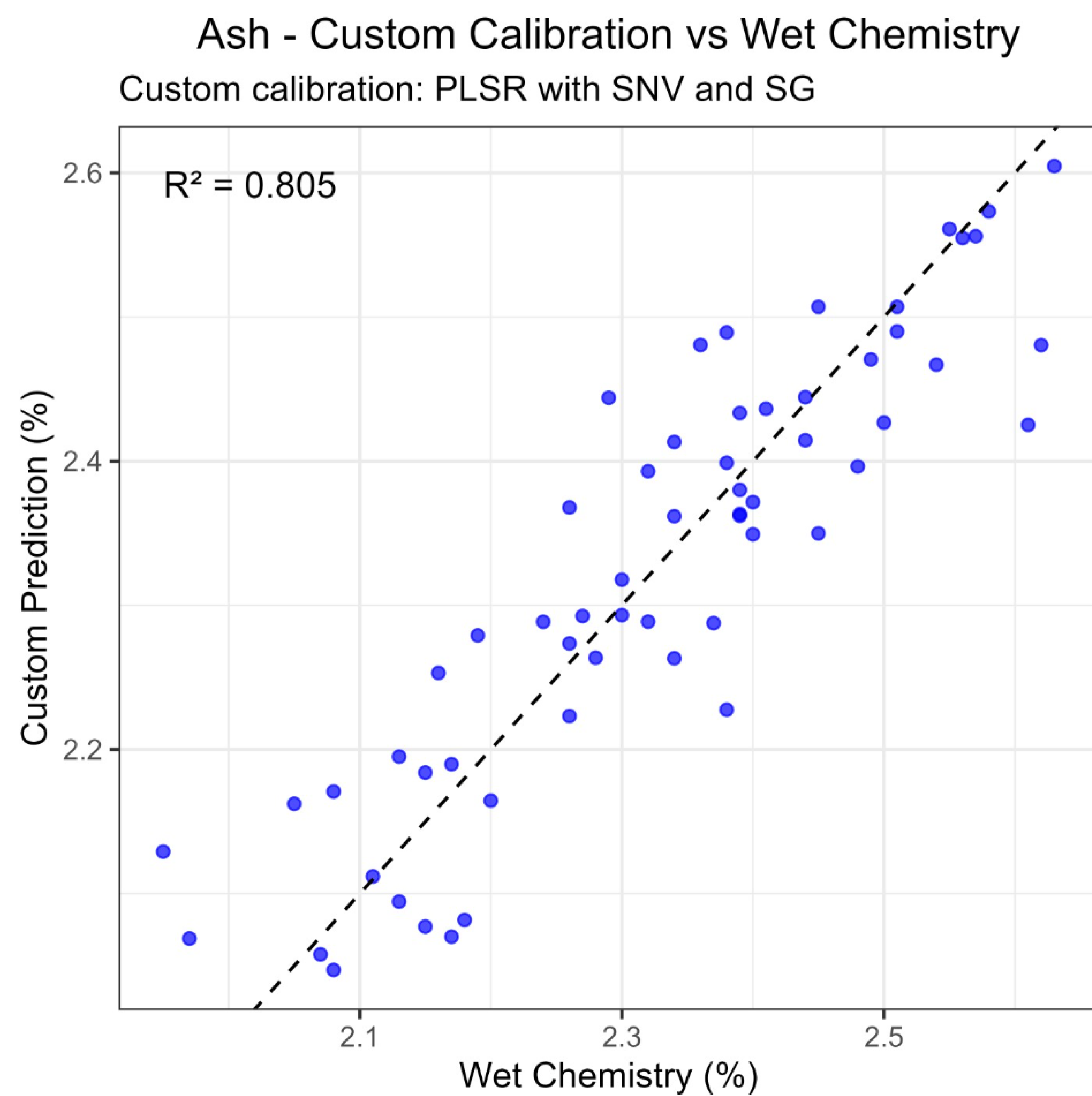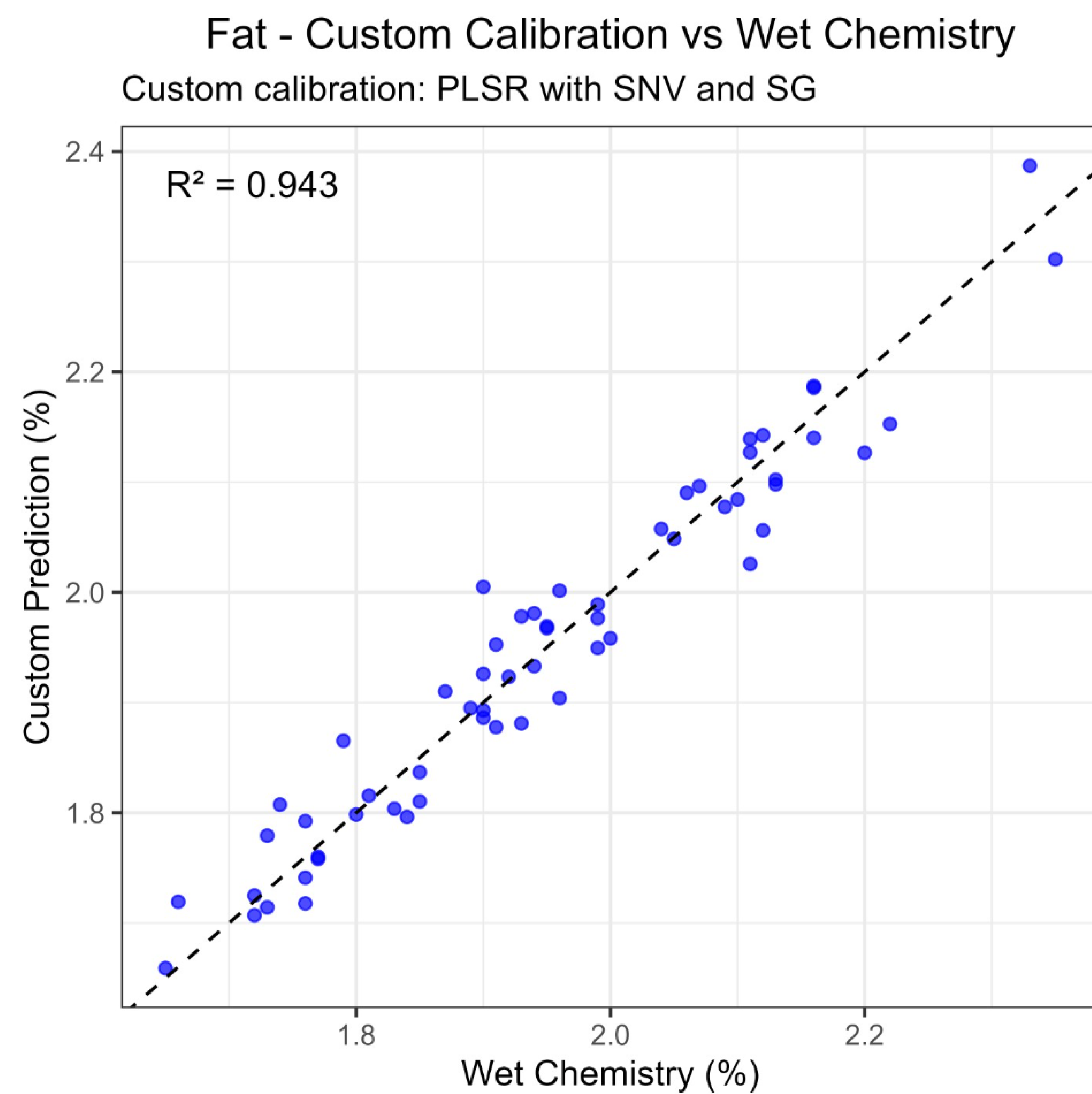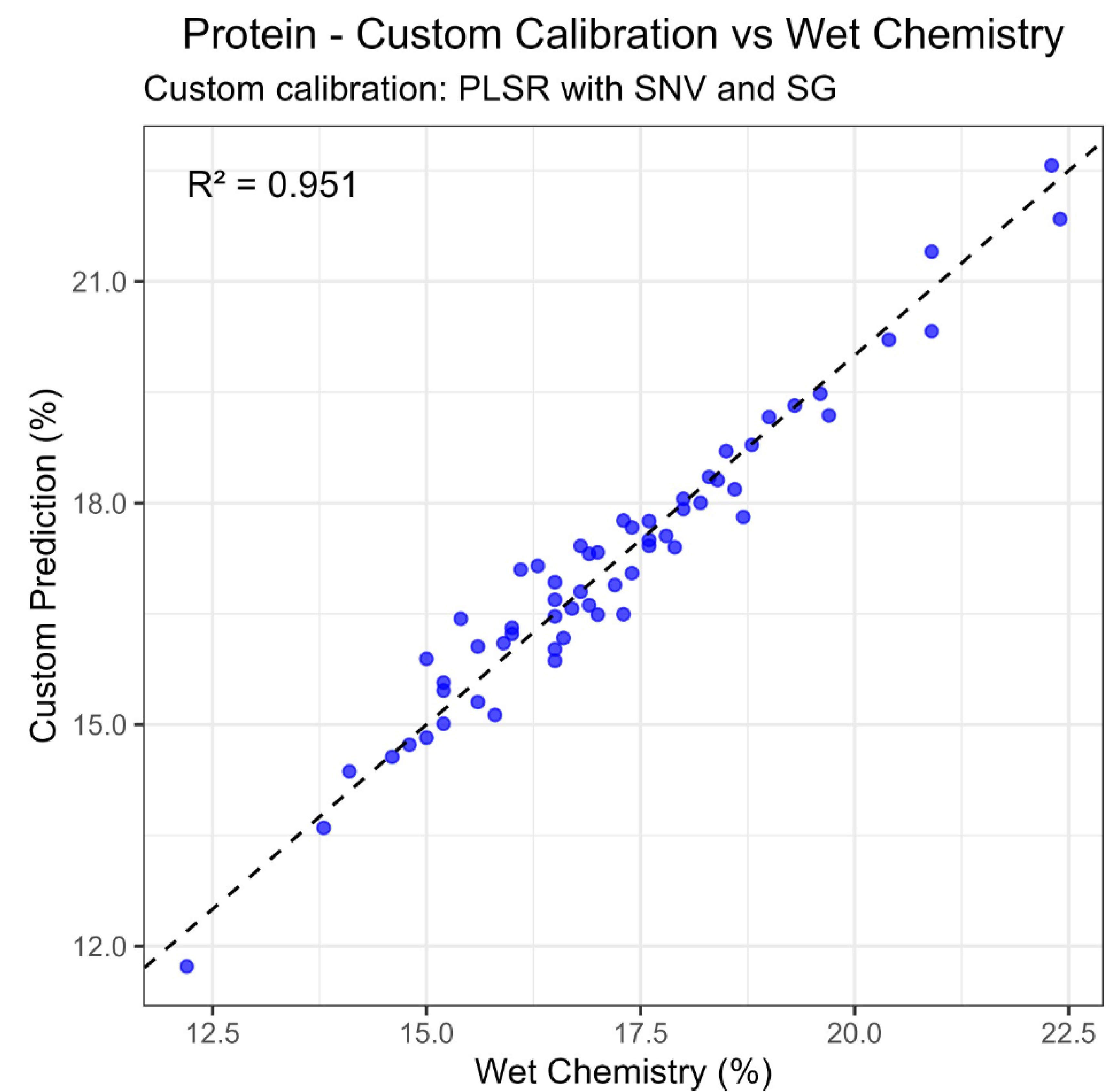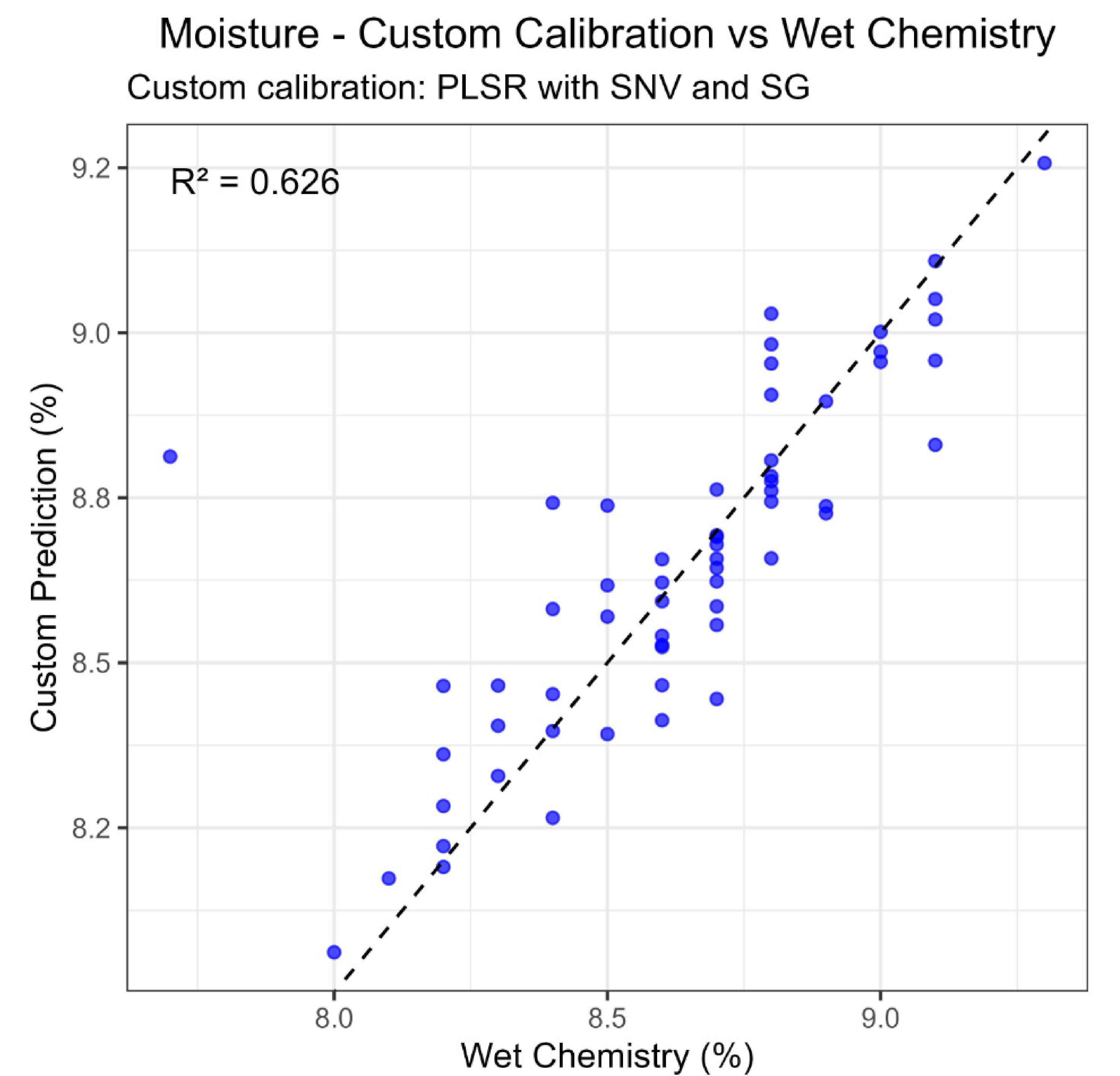

Supplement: Supplementary file 1 — Supplementary Material 1 (ZIP 5.74 MB) [file 11032_2026_1673_MOESM1_ESM.zip › Supplementary Material/ESM_7.pdf]
