# Supplementary material for: Wild barley cytoplasms reduce grain weight plasticity, with environment-dependent cytonuclear epistasis at the ari-e locus
Source: Mol Breed. 2026 May 19;46(6):48. doi: 10.1007/s11032-026-01673-6 (PMC13187085; doi:10.1007/s11032-026-01673-6)
Supplement: Supplementary file 3 — Supplementary Material 3 (PDF 6.25 MB) [file 11032_2026_1673_MOESM3_ESM.pdf]

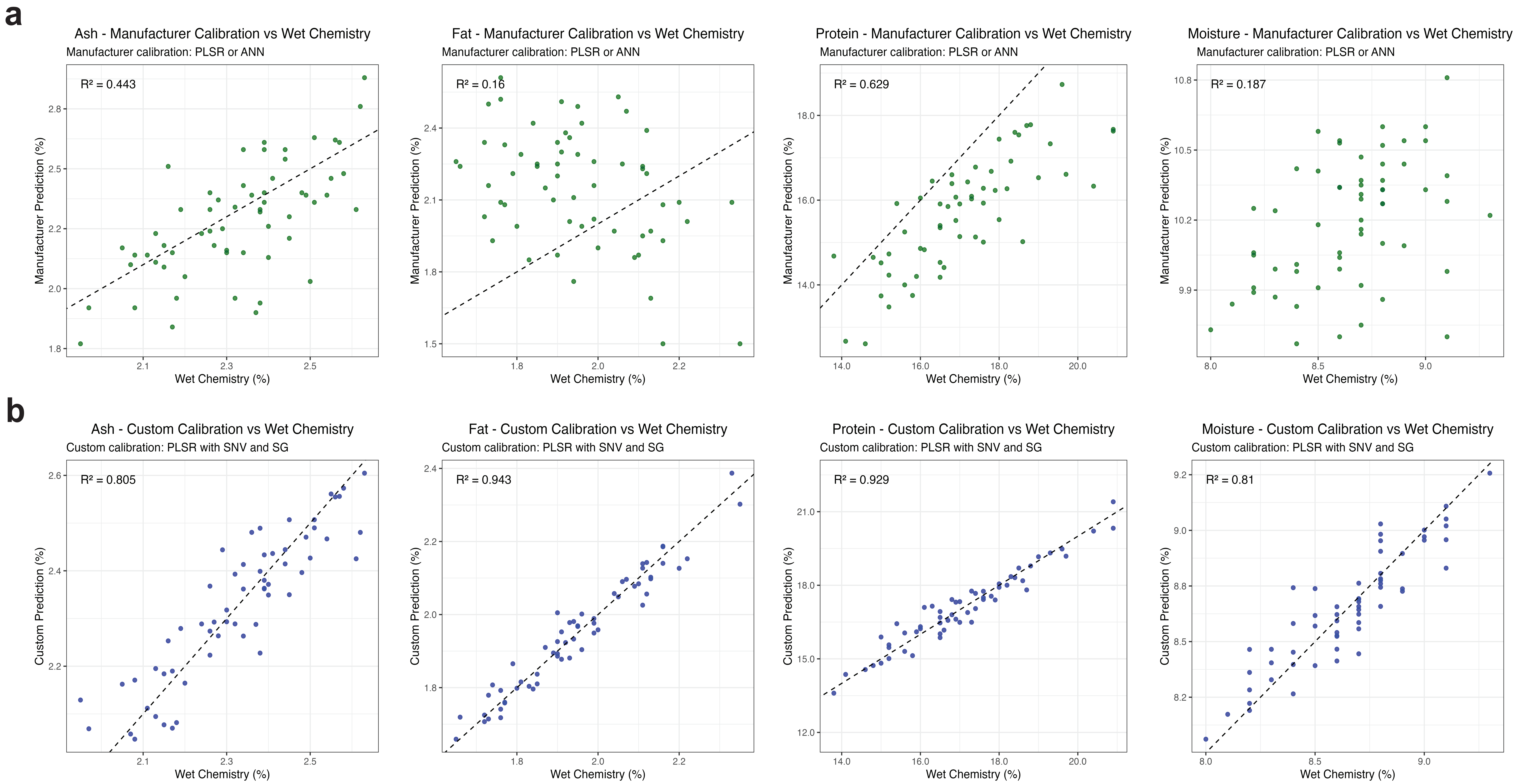
a
Ash - Manufacturer Calibration vs Wet Chemistry
Manufacturer calibration: PLSR or ANN
R² = 0.443
Fat - Manufacturer Calibration vs Wet Chemistry
Manufacturer calibration: PLSR or ANN
R² = 0.16
Protein - Manufacturer Calibration vs Wet Chemistry
Manufacturer calibration: PLSR or ANN
R² = 0.629
Moisture - Manufacturer Calibration vs Wet Chemistry
Manufacturer calibration: PLSR or ANN
R² = 0.187
Manufacturer Prediction (%)
Wet Chemistry (%)
b
Ash - Custom Calibration vs Wet Chemistry
Custom calibration: PLSR with SNV and SG
R² = 0.805
Fat - Custom Calibration vs Wet Chemistry
Custom calibration: PLSR with SNV and SG
R² = 0.943
Protein - Custom Calibration vs Wet Chemistry
Custom calibration: PLSR with SNV and SG
R² = 0.929
Moisture - Custom Calibration vs Wet Chemistry
Custom calibration: PLSR with SNV and SG
R² = 0.81
Custom Prediction (%)
Wet Chemistry (%)
